# Supplementary material for: Prevalence and Determinants of COVID-19 Vaccine Acceptance in South East Asia: A Systematic Review and Meta-Analysis of 1,166,275 Respondents
Source: Trop Med Infect Dis. 2022 Nov 9;7(11):361. doi: 10.3390/tropicalmed7110361 (PMC9696885; doi:10.3390/tropicalmed7110361)
Supplement: Supplementary file 1 [file tropicalmed-07-00361-s001.zip › tropicalmed-1948369-supplementary.pdf]

Table S1. Medical subject heading (MeSH) terms and keywords used in each database.

Database	Medical subject heading	The number of studies found
Pubmed	("covid 19"[All Fields] OR "covid 19"[MeSH Terms] OR "covid 19 vaccines"[All Fields] OR "covid 19 vaccines"[MeSH Terms] OR "covid 19 serotherapy"[All Fields] OR "covid 19 serotherapy"[Supplementary Concept] OR "covid 19 nucleic acid testing"[All Fields] OR "covid 19 nucleic acid testing"[MeSH Terms] OR "covid 19 serological testing"[All Fields] OR "covid 19 serological testing"[MeSH Terms] OR "covid 19 testing"[All Fields] OR "covid 19 testing"[MeSH Terms] OR "sars cov 2"[All Fields] OR "sars cov 2"[MeSH Terms] OR "severe acute respiratory syndrome coronavirus 2"[All Fields] OR "ncov"[All Fields] OR "2019 ncov"[All Fields] OR (("coronavirus"[MeSH Terms] OR "coronavirus"[All Fields] OR "cov"[All Fields]) AND 2019/11/01:3000/12/31[Date - Publication]) OR ("sars cov 2"[MeSH Terms] OR "sars cov 2"[All Fields] OR "sars cov 2"[All Fields])) AND ((("vaccin"[Supplementary Concept] OR "vaccin"[All Fields] OR "vaccination"[MeSH Terms] OR "vaccination"[All Fields] OR "vaccinable"[All Fields] OR "vaccinal"[All Fields] OR "vaccinate"[All Fields] OR "vaccinated"[All Fields] OR "vaccinates"[All Fields] OR "vaccinating"[All Fields] OR "vaccinations"[All Fields] OR "vaccination s"[All Fields] OR "vaccinator"[All Fields] OR "vaccinators"[All Fields] OR "vaccine s"[All Fields] OR "vaccined"[All Fields] OR "vaccines"[MeSH Terms] OR "vaccines"[All Fields] OR "vaccine"[All Fields] OR "vaccins"[All Fields]) AND ("accept"[All Fields] OR "acceptabilities"[All Fields] OR "acceptability"[All Fields] OR "acceptable"[All Fields] OR "acceptably"[All Fields] OR "acceptance"[All Fields] OR "acceptances"[All Fields] OR "acceptation"[All Fields] OR "accepted"[All Fields] OR "accepter"[All Fields] OR "accepters"[All Fields] OR "accepting"[All Fields] OR "accepts"[All Fields])) OR (("vaccin"[Supplementary Concept] OR "vaccin"[All Fields] OR "vaccination"[MeSH Terms] OR "vaccination"[All Fields] OR "vaccinable"[All Fields] OR "vaccinal"[All Fields] OR "vaccinate"[All Fields] OR "vaccinated"[All Fields] OR "vaccinates"[All Fields] OR "vaccinating"[All Fields] OR "vaccinations"[All Fields] OR "vaccination s"[All Fields] OR "vaccinator"[All Fields] OR "vaccinators"[All Fields] OR "vaccine s"[All Fields] OR "vaccined"[All Fields] OR "vaccines"[MeSH Terms] OR "vaccines"[All Fields] OR "vaccine"[All Fields] OR "vaccins"[All Fields]) AND ("hesitance"[All Fields] OR "hesitancies"[All Fields] OR "hesitancy"[All Fields] OR "hesitant"[All Fields] OR "hesitate"[All Fields] OR "hesitated"[All Fields] OR "hesitating"[All Fields] OR "hesitation"[All Fields] OR "hesitations"[All Fields])) OR ("vaccination refusal"[MeSH Terms] OR ("vaccination"[All Fields] AND "refusal"[All Fields]) OR "vaccination refusal"[All Fields] OR ("vaccine"[All Fields] AND "refusal"[All Fields]) OR "vaccine refusal"[All Fields]) OR (("vaccin"[Supplementary Concept] OR "vaccin"[All Fields] OR "vaccination"[MeSH Terms] OR "vaccination"[All Fields] OR "vaccinable"[All Fields] OR "vaccinal"[All Fields] OR "vaccinate"[All Fields] OR "vaccinated"[All Fields] OR "vaccinates"[All Fields] OR "vaccinating"[All Fields] OR "vaccinations"[All Fields] OR "vaccination s"[All Fields] OR "vaccinator"[All Fields] OR "vaccinators"[All Fields] OR "vaccine s"[All Fields] OR "vaccined"[All Fields] OR "vaccines"[MeSH Terms] OR "vaccines"[All Fields] OR "vaccine"[All Fields] OR "vaccins"[All Fields]) AND ("uptake"[All Fields] OR "uptakes"[All Fields] OR "uptaking"[All Fields]))) AND ("asia, southeastern"[MeSH Terms] OR ("asia"[All Fields] AND "southeastern"[All Fields]) OR "southeastern asia"[All Fields] OR ("southeast"[All Fields] AND "asia"[All Fields]) OR "southeast asia"[All Fields] OR ("brunei"[MeSH Terms] OR "brunei"[All Fields] OR ("brunei"[All Fields] AND "darussalam"[All Fields]) OR "brunei darussalam"[All Fields]) OR ("indonesia"[MeSH Terms] OR "indonesia"[All Fields] OR "indonesia s"[All Fields] OR "indonesias"[All Fields]) OR ("singapore"[MeSH Terms] OR "singapore"[All Fields] OR "singapore s"[All Fields]) OR ("malaysia"[MeSH Terms] OR "malaysia"[All Fields] OR "malaysia s"[All Fields]) OR ("timor leste"[MeSH Terms] OR "timor leste"[All Fields] OR ("timor"[All Fields] AND "leste"[All Fields]) OR "timor leste"[All Fields]) OR ("philippine"[All Fields]	305

	OR "philippines"[MeSH Terms] OR "philippines"[All Fields]) OR ("myanmar"[MeSH Terms] OR "myanmar"[All Fields] OR "myanmar s"[All Fields] OR "myanmars"[All Fields]) OR ("cambodia"[MeSH Terms] OR "cambodia"[All Fields] OR "cambodia s"[All Fields]) OR ("thailand"[MeSH Terms] OR "thailand"[All Fields] OR "thailand s"[All Fields]) OR ("vietnam"[MeSH Terms] OR "vietnam"[All Fields] OR "vietnam s"[All Fields]) OR ("laos"[MeSH Terms] OR "laos"[All Fields]))	
Medline	(((((((((((((((COVID-19) OR SARS-CoV-2) AND vaccine acceptance) OR vaccine hesitancy) OR vaccine refusal) OR vaccine uptake) AND southeast asia) OR brunei darussalam) OR indonesia) OR singapore) OR malaysia) OR timor leste) OR philippines) OR myanmar) OR cambodia) OR thailand) OR vietnam) OR laos Filters: Publication date from 2020/01/01 to 2022/12/31	164955
Cochrane Central Register of Controlled Trials (CENTRAL)	(COVID-19 Vaccine Acceptance):ti,ab,kw AND (Indonesia):ti,ab,kw OR (Laos):ti,ab,kw OR (Brunei):ti,ab,kw OR (Singapore):ti,ab,kw	1912
Google Scholar	"COVID 19 Vaccine Acceptance" -Turkey -France -Nigeria -Jordan -United -Kingdom -UK -China -United -States -USA -Africa -Taiwan -Europe -Finland -Lebanon	120
	"COVID 19 Vaccine Hesitancy" -Brazil -US -Israel -Japan -Korea -Canada -Turkey -France -Nigeria -Jordan -United -Kingdom -UK -China -United -States -USA -Africa -Taiwan -India -Europe -Finland -Lebanon	81
	"COVID 19 Vaccine refusal" -Brazil -US -Israel -Japan -Korea -Canada -Turkey -France -Nigeria -Jordan -United -Kingdom -UK -China -United -States -USA -Africa -Taiwan -India -Europe -Finland -Lebanon	4
	"COVID 19 Vaccine uptake" -Bangladesh -Brazil -US -Israel -Japan -Korea -Canada -Turkey -France -Nigeria -Jordan -United -Kingdom -UK -China -United -States -USA -Africa -Taiwan -India -Europe -Finland -Lebanon	26
MedRxiv & BioRxiv	"covid-19 vaccine acceptance in southeast asia" and posted between "01 Jan, 2020 and 31 Dec, 2022"	134
	"covid-19 vaccine acceptance in Brunei" and posted between "01 Jan, 2020 and 31 Dec, 2022"	15
	"covid-19 vaccine acceptance in myanmar" and posted between "01 Jan, 2020 and 31 Dec, 2022"	31
	"covid-19 vaccine acceptance in cambodia" and posted between "01 Jan, 2020 and 31 Dec, 2022"	35
	"covid-19 vaccine acceptance in indonesia" and posted between "01 Jan, 2020 and 31 Dec, 2022"	157
	"covid-19 vaccine acceptance in laos" and posted between "01 Jan, 2020 and 31 Dec, 2022"	37
	"covid-19 vaccine acceptance in malaysia" and posted between "01 Jan, 2020 and 31 Dec, 2022"	148
	"covid-19 vaccine acceptance in Singapore" and posted between "01 Jan, 2020 and 31 Dec, 2022"	346
	"covid-19 vaccine acceptance in thailand" and posted between "01 Jan, 2020 and 31 Dec, 2022"	183
	"covid-19 vaccine acceptance in vietnam" and posted between "01 Jan, 2020 and 31 Dec, 2022"	140
Science Direct	covid-19 vaccine acceptance OR covid-19 vaccine hesitancy OR covid-19 vaccine refusal	4225
Indonesian Scientific Journal Database	Vaksin COVID-19	4
	COVID-19 Vaccine	2
Neliti	Vaksin COVID-19	74
	COVID-19 vaccine	179
Indonesia One Search	Vaksin COVID-19	176
	COVID-19 Vaccine Indonesia	60
ThaiJo	COVID-19 Vaccine Acceptance	3

	COVID-19 Vaccination Hesitancy	3
	COVID-19 vaccination uptake	3
Thai-Journal Citation Index Centre	COVID-19 Vaccination	3

Table S2. Vaccine rollout date for each country [1–8].

Country	Start of vaccine rollout
Indonesia[1]	13th January 2021
Malaysia[2]	24th February 2021
Singapore[3]	30th December 2020
Thailand[4]	28th February 2021
The Philippines[5]	1st March 2021
Brunei Darussalam[6]	3rd April 2021
East Timor[7]	14th June 2021
Laos[6]	2nd April 2021
Cambodia[6]	10th February 2021
Vietnam[8]	8th March 2021
Myanmar[6]	27th January 2021

Table S3. Newcastle Ottawa Scale of each study [9–114].

Author (year)	Newcastle Ottawa Scale				
	Selection	Comparability	Outcome	Total	Classification
Ardiningsih & Kardiwinata (2021)[9]	1	0	2	3	Poor
Aw (2022)[10]	3	2	2	7	Good
Bautista Jr. (2021)[11]	1	0	2	3	Poor
Bono* (2021)[12]	3	1	2	6	Moderate
Boon-Itt (2021)[13]	3	0	2	5	Moderate
Boontho (2022)[14]	2	1	2	5	Moderate
Cahapay (2022)[15]	4	1	2	7	Good
Caple (2022)[16]	4	1	2	7	Good
Chen (2022)[17]	2	0	2	4	Moderate
Chew (2021)[18]	3	2	2	7	Good
Crespo (2021)[19]	2	0	1	3	Poor

Davis (2022)[20]	3	2	2	7	Good
de Figueiredo (2021)[21]	5	2	2	9	Good
Duong (2021)[22]	5	2	2	9	Good
Elnaem (2021)[23]	4	1	2	7	Good
Enea (2022)[24]	5	2	2	9	Good
Ginting (2021)[25]	1	0	1	2	Poor
Griva (2021)[26]	3	2	2	7	Good
Ha (2021)[27]	1	0	1	2	Poor
Hadiwijaya (2021)[28]	1	0	1	2	Poor
Halu (2022)[29]	0	0	1	1	Poor
Hanvivattanakul (2022)[30]	1	0	1	2	Poor
Harapan (2020)[31]	3	2	2	7	Good
Hartigan-Go (2021)[32]	5	2	2	9	Good
Humanity & Inclusion (2021)[33]	N/A	N/A	N/A	N/A	
Huynh (2021)[34]	2	1	2	5	Moderate
Huynh (2021)[35]	3	1	2	6	Moderate
Ichsan (2021)[36]	0	0	1	1	Poor
Internasional Organization for Migration (IOM)[37]	5	2	2	9	Good
Jafar (2022)[38]	4	2	2	8	Good
Juin (2022)[39]	2	2	2	6	Moderate
Jukkrit (2021)[40]	3	1	1	5	Moderate
Kementerian Kesihatan Malaysia (2020)[41]	N/A	N/A	N/A	N/A	
Kerekes (2021)[42]	3	1	2	6	Moderate
Khuc (2021)[43]	3	1	3	6	Good
Khuc (2021)[44]	0	1	1	2	Poor
Kitro (2021)[45]	4	2	2	8	Good
Kitro (2022)[46]	3	2	2	7	Good
Koesnoe (2022)[47]	4	2	2	8	Good
Koh (2022)[48]	4	2	2	8	Good
Koh (2022)[49]	4	2	3	9	Good
Lansford (2022)[50]	3	2	2	7	Good
Lasmita (2021)[51]	3	2	2	7	Good
Lau (2021)[52]	4	2	2	8	Good
Lazarus (2021)[53]	4	2	1	7	Good
Lazarus (2022)[54]	5	2	2	9	Good

Le (2022)[55]	4	2	2	8	Good
Le (2022)[56]	4	2	2	8	Good
Le An (2021)[57]	4	0	2	6	Moderate
Le An (2021)[58]	4	0	2	6	Moderate
Li (2022)[59]	5	2	2	9	Good
Lim (2021)[60]	4	2	3	9	Good
Lin (2022)[61]	3	2	1	6	Moderate
Marzo (2022)[62]	3	1	2	6	Moderate
Marzo (2022)[63]	2	1	2	5	Moderate
Ministry of Health of Brunei Darussalam (2021)[64]	N/A	N/A	N/A	N/A	
Mohamed (2021)[65]	3	1	2	6	Moderate
Ng (2022)[66]	3	1	2	6	Moderate
Ng (2022)[67]	3	1	2	6	Moderate
Nguyen (2021)[8]	4	2	2	8	Good
Nguyen (2021)[68]	2	1	2	5	Moderate
Nhu (2021)[69]	1	0	1	2	Poor
Noushad (2021)[70]	2	1	2	5	Moderate
OCTA Research Group (2021)[71]	N/A	N/A	N/A	N/A	
OCTA Research Group (2021)[72]	N/A	N/A	N/A	N/A	
Octavius (2022)[73]	2	1	2	5	Moderate
Pagador (2022)[74]	3	2	2	7	Good
Pairat & Phaloprakarn (2022)[75]	4	1	2	7	Good
Payaprom (2022)[76]	4	2	2	8	Good
Pheerapanyawaranun (2022)[4]	3	2	2	7	Good
Prasert (2022)[77]	4	2	2	8	Good
Puspitasari (2022)[78]	2	2	2	6	Moderate
Putri (2021)[79]	3	2	2	7	Good
Rani (2022)[80]	5	2	2	9	Good
Rizki (2021)[81]	3	2	2	7	Good
Rozek (2021)[82]	2	2	3	7	Good
Saida (2022)[83]	3	1	2	6	Moderate
Shah (2022)[84]	4	2	2	8	Good
Sidarta (2022)[85]	5	2	2	9	Good
Siewchaisakul (2022)[86]	4	2	2	8	Good
Simanjorang (2022)[87]	3	1	2	6	Moderate
Sirikalyanpaiboon (2021)[88]	4	2	2	8	Good

Skjefte (2021)[89]	3	2	2	7	Good
Syed Alwi (2021)[90]	5	2	2	9	Good
Tan (2022)[91]	1	1	2	4	Moderate
Tan (2022)[92]	2	2	2	6	Moderate
Tan (2022)[93]	3	2	2	7	Good
Tan (2022)[94]	2	1	1	4	Moderate
Thanapluetiwong (2021)[95]	3	1	2	6	Moderate
The Asia Foundation (2021)[7]	2	2	1	5	Moderate
The Asia Foundation (2021)[96]	2	2	1	5	Moderate
The Asia Foundation (2021)[97]	2	2	1	5	Moderate
The Asia Foundation (2021)[98]	2	2	1	5	Moderate
Thi Xuan Hoang (2022)[99]	3	1	2	6	Moderate
UNDP Philippines (2021)[100]	N/A	N/A	N/A	N/A	
UNICEF (2020)[101]	3	2	2	7	Good
Utami (2022)[102]	3	1	2	6	Moderate
Vaghefi (2021)[103]	N/A	N/A	N/A	N/A	
Wen (2020)[104]	1	0	1	2	Poor
Wirawan (2021)[105]	2	1	2	5	Moderate
Wirawan (2022)[106]	3	2	2	7	Good
Wong (2022)[107]	3	2	2	7	Good
Wong (2020)[108]	2	1	2	5	Moderate
Wong (2021)[109]	3	2	2	7	Good
Wong (2022)[110]	2	1	2	5	Moderate
Wong (2022)[111]	3	1	2	6	Moderate
Wong (2022)[112]	2	1	2	5	Moderate
World Vision International (2021)[113]	3	0	2	5	Moderate
Yoda (2022)[114]	3	0	2	5	Moderate

Table S4. Assessment of risk of bias using Joanna Briggs Institute (JBI) Checklist [9–114].

Author (year)	JBI 1	JBI 2	JBI 3	JBI 4	JBI 5	JBI 6	JBI 7	JBI 8	Total JBI score	Overall risk of bias
Ardiningsih & Kardiwinata (2021)[9]	U	N	U	Y	N	N	U	N	1	Low risk
Aw (2022)[10]	Y	Y	Y	U	Y	U	Y	Y	2	Low risk

Bautista Jr. (2021)[11]	Y	N	N	B	N	N	Y	Y	5	Moderate risk
Bono* (2021)[12]	U	N	U	U	U	N	U	Y	7	High risk
Boon-Itt (2021)[13]	U	N	U	U	N	N	Y	Y	6	Moderate risk
Boontho (2022)[14]	Y	Y	U	U	Y	U	Y	Y	4	Moderate risk
Cahapay (2022)[15]	U	Y	Y	Y	N	N	Y	Y	3	Low risk
Caple (2022)[16]	Y	N	Y	Y	U	U	Y	Y	3	Low risk
Chen (2022)[17]	N	N	U	Y	N	N	Y	Y	5	Moderate risk
Chew (2021)[18]	N	N	Y	U	Y	Y	Y	Y	3	Low risk
Crespo (2021)[19]	N	N	U	U	N	N	Y	N	7	High risk
Davis (2022)[20]	Y	N	U	Y	Y	Y	Y	Y	2	Low risk
de Figueiredo (2021)[21]	Y	U	Y	Y	Y	Y	Y	Y	1	Low risk
Duong (2021)[22]	Y	Y	Y	Y	Y	U	Y	N	2	Low risk
Elnaem (2021)[23]	Y	N	Y	Y	U	U	Y	Y	3	Low risk
Enea (2022)[24]	U	U	Y	Y	Y	Y	Y	Y	2	Low risk
Ginting (2021)[25]	U	U	N	N	N	Y	Y	U	6	Moderate risk
Griva (2021)[26]	Y	Y	Y	Y	Y	Y	Y	Y	0	Low risk
Ha (2021)[27]	N	N	U	Y	U	N	N	N	7	High risk
Hadiwijaya (2021)[28]	U	U	U	N	N	U	Y	N	7	High risk
Halu (2022)[29]	N	U	N	N	N	U	U	U	8	High risk
Hanvivattanakul (2022)[30]	U	U	Y	U	U	U	U	N	7	High risk
Harapan (2020)[31]	Y	U	Y	N	Y	Y	Y	Y	2	Low risk
Hartigan-Go (2021)[32]	Y	N	Y	Y	Y	Y	Y	Y	1	Low risk
Humanity & Inclusion (2021)[33]	N/A	N/A	N/A	N/A	N/A	N/A	N/A	N/A		
Huynh (2021)[34]	U	Y	Y	N	U	U	U	Y	6	Moderate risk
Huynh (2021)[35]	Y	U	Y	N	N	U	Y	Y	4	Moderate risk
Ichsan (2021)[36]	N	N	U	U	N	U	U	Y	7	High risk
Internasional Organization for Migration (IOM)[37]	Y	Y	Y	Y	Y	Y	Y	Y	0	Low risk
Jafar (2022)[38]	N	Y	Y	Y	N	N	Y	Y	3	Low risk
Juin (2022)[39]	U	Y	U	N	N	U	Y	Y	5	Moderate risk
Jukkrit (2021)[40]	U	Y	Y	U	N	U	Y	N	5	Moderate risk
Kementerian Kesihatan Malaysia (2020)[41]	N/A	N/A	N/A	N/A	N/A	N/A	N/A	N/A		
Kerekes (2021)[42]	Y	N	Y	Y	N	U	Y	U	4	Moderate risk
Khuc (2021)[43]	U	N	Y	Y	U	U	U	Y	5	Moderate risk
Khuc (2021)[44]	N	U	Y	N	U	U	U	N	7	High risk

Kitro (2021)[45]	Y	U	Y	N	Y	Y	Y	Y	2	Low risk
Kitro (2022)[46]	Y	U	Y	N	N	Y	Y	Y	3	Low risk
Koesnoe (2022)[47]	Y	Y	Y	Y	N	N	Y	Y	2	Low risk
Koh (2022)[48]	Y	Y	Y	Y	Y	N	Y	Y	1	Low risk
Koh (2022)[49]	Y	Y	Y	Y	Y	Y	Y	Y	0	Low risk
Lansford (2022)[50]	Y	Y	U	Y	Y	Y	Y	Y	1	Low risk
Lasmita (2021)[51]	N	Y	Y	Y	N	N	Y	Y	3	Low risk
Lau (2021)[52]	N	Y	Y	Y	N	N	Y	Y	3	Low risk
Lazarus (2021)[53]	N	Y	Y	Y	N	N	Y	Y	3	Low risk
Lazarus (2022)[54]	Y	U	Y	Y	Y	Y	Y	Y	1	Low risk
Le (2022)[55]	N	Y	Y	Y	N	N	Y	Y	3	Low risk
Le (2022)[56]	Y	Y	U	Y	Y	Y	U	Y	2	Low risk
Le An (2021)[57]	U	N	U	Y	N	N	U	Y	6	Moderate risk
Le An (2021)[58]	U	U	U	Y	N	N	Y	Y	5	Moderate risk
Li (2022)[59]	Y	Y	Y	Y	Y	Y	Y	Y	0	Low risk
Lim (2021)[60]	N	Y	Y	Y	Y	Y	Y	Y	1	Low risk
Lin (2022)[61]	Y	N	Y	Y	Y	N	Y	Y	2	Low risk
Marzo (2022)[62]	U	U	U	Y	U	U	U	Y	6	Moderate risk
Marzo (2022)[63]	U	N	U	Y	U	U	Y	Y	5	Moderate risk
Ministry of Health of Brunei Darussalam (2021)[64]	N/A	N/A	N/A	N/A	N/A	N/A	N/A	N/A		
Mohamed (2021)[65]	U	N	Y	Y	N	N	Y	Y	4	Moderate risk
Ng (2022)[66]	U	N	Y	Y	U	U	U	Y	5	Moderate risk
Ng (2022)[67]	Y	N	Y	Y	U	U	Y	Y	3	Low risk
Nguyen (2021)[8]	Y	U	U	U	Y	Y	Y	Y	3	Low risk
Nguyen (2021)[68]	Y	U	U	Y	N	N	Y	Y	4	Moderate risk
Nhu (2021)[69]	N	N	U	U	N	N	N	Y	1	Low risk
Noushad (2021)[70]	N	U	Y	N	N	N	U	Y	6	Moderate risk
OCTA Research Group (2021)[71]	N/A	N/A	N/A	N/A	N/A	N/A	N/A	N/A		
OCTA Research Group (2021)[72]	N/A	N/A	N/A	N/A	N/A	N/A	N/A	N/A		
Octavius (2022)[73]	Y	N	U	U	N	N	U	Y	6	Moderate risk
Pagador (2022)[74]	Y	N	Y	Y	N	N	U	Y	4	Moderate risk
Pairat & Phaloprakarn (2022)[75]	Y	Y	Y	Y	U	U	Y	Y	2	Low risk
Payaprom (2022)[76]	Y	N	Y	Y	Y	N	Y	Y	2	Low risk

Pheerapanyawaranun (2022)[4]	Y	Y	Y	Y	Y	Y	Y	Y	0	Low risk
Prasert (2022)[77]	Y	Y	Y	Y	Y	Y	Y	Y	0	Low risk
Puspitasari (2022)[78]	N	Y	Y	Y	N	N	Y	Y	3	Low risk
Putri (2021)[79]	N	Y	Y	Y	N	N	Y	Y	3	Low risk
Rani (2022)[80]	Y	Y	Y	Y	N	N	Y	Y	2	Low risk
Rizki (2021)[81]	N	Y	Y	Y	N	N	Y	Y	3	Low risk
Rozek (2021)[82]	Y	Y	Y	Y	Y	Y	Y	Y	0	Low risk
Saida (2022)[83]	N	Y	Y	Y	N	N	Y	Y	3	Low risk
Shah (2022)[84]	Y	U	Y	U	Y	U	Y	Y	3	Low risk
Sidarta (2022)[85]	Y	Y	Y	Y	Y	Y	Y	Y	0	Low risk
Siewchaisakul (2022)[86]	Y	Y	Y	Y	N	N	Y	Y	2	Low risk
Simanjorang (2022)[87]	Y	Y	U	Y	N	N	Y	Y	3	Low risk
Sirikalyanpaiboon (2021)[88]	N	Y	Y	Y	N	N	Y	Y	3	Low risk
Skjefte (2021)[89]	N	Y	Y	Y	N	N	Y	Y	3	Low risk
Syed Alwi (2021)[90]	N	Y	Y	Y	N	N	Y	Y	3	Low risk
Tan (2022)[91]	U	N	U	Y	U	N	Y	Y	5	Moderate risk
Tan (2022)[92]	N	N	Y	Y	Y	U	Y	Y	3	Low risk
Tan (2022)[93]	Y	N	Y	Y	Y	U	Y	Y	2	Low risk
Tan (2022)[94]	U	N	U	U	Y	N	Y	U	6	Moderate risk
Thanapluetiwong (2021)[95]	Y	Y	Y	Y	U	N	U	Y	3	Low risk
The Asia Foundation (2021)[7]	Y	Y	U	Y	Y	U	U	N	4	Moderate risk
The Asia Foundation (2021)[96]	Y	Y	U	Y	Y	U	U	N	4	Moderate risk
The Asia Foundation (2021)[97]	Y	Y	U	Y	Y	U	U	N	4	Moderate risk
The Asia Foundation (2021)[98]	Y	Y	U	Y	Y	U	U	N	4	Moderate risk
Thi Xuan Hoang (2022)[99]	U	Y	Y	N	Y	U	U	Y	4	Moderate risk
UNDP Philippines (2021)[100]	N/A	N/A	N/A	N/A	N/A	N/A	N/A	N/A		
UNICEF (2020)[101]	N	N	U	U	Y	Y	Y	N	5	Moderate risk
Utami (2022)[102]	U	N	Y	Y	N	N	Y	Y	4	Moderate risk
Vaghefi (2021)[103]	N/A	N/A	N/A	N/A	N/A	N/A	N/A	N/A		
Wen (2020)[104]	U	N	U	U	N	U	U	N	8	High risk
Wirawan (2021)[105]	Y	N	U	U	N	U	U	Y	6	Moderate risk
Wirawan (2022)[106]	Y	N	Y	Y	N	N	Y	Y	3	Low risk

Wong (2022)[107]	Y	N	Y	Y	N	N	Y	Y	3	Low risk
Wong (2020)[108]	Y	N	Y	U	N	N	U	Y	5	Moderate risk
Wong (2021)[109]	Y	U	Y	U	N	N	Y	Y	5	Moderate risk
Wong (2022)[110]	Y	N	Y	Y	N	N	Y	Y	3	Low risk
Wong (2022)[111]	Y	N	N	Y	N	N	Y	Y	4	Moderate risk
Wong (2022)[112]	N	N	U	Y	U	N	Y	Y	5	Moderate risk
World Vision International (2021)[113]	N	N	N	N	N	N	Y	Y	6	Moderate risk
Yoda (2022)[114]	Y	N	U	Y	N	N	Y	Y	4	Moderate risk

Y = Yes; U= Unclear; N = No.

Table S5. Notable exclusions and their reasons [115–141].

Authors	Reasons for exclusions
Bono (2021)[115]	Utilised the same database(s)
Chen (2022)[116]	
Duong (2022)[117]	
Harapan (2020)[118]	
Jafar (2022)[119]	
Leigh (2022)[120]	
Marzo (2022)[121]	
Yanto (2021)[122]	
Zhang (2021)[123]	
Liew (2022)[124]	N<50 samples
Lim (2022)[125]	
Landicho-Guevarra (2021)[126]	Not COVID-19 vaccines
Migrino Jr (2020)[127]	
Musa (2019)[128]	
Shaaban (2022)[129]	No primary data on vaccine acceptance (social media studies)
Duong & Antriyandarti (2022)[130]	
Faturohman (2021)[131]	
Leesawat (2022)[132]	
Mangla (2021)[133]	
Mueangpoon (2021)[134]	
Sitrait (2022)[135]	
Susilawaty (2021)[136]	
Teh (2022)[137]	

The World Bank (2021)[138]	No data on vaccine acceptance (did not provide the rate, focusing solely on structured equation model analysis or the theories involved), only focusing solely on vaccine hesitance/refusal or solely studying willingness to pay for COVID-19 vaccines
Theodorea (2021)[139]	
Wee (2021)[140]	
Zagefka (2022)[141]	

Table S6. Subgroups of COVID-19 acceptance prevalence.

Subgroups	Prevalence (95 confidence interval) (%)	Weight (%)	I^2 (%)	p-value
	Types of paper			
Peer-reviewed journals (N=111)	73 (70–75)	82.22	99.79	<0.001
Grey literature (N=24)	65 (60–70)	17.78	99.95	<0.001
Booster or not booster				
Booster (N=6)	58 (44–71)	4.48	99.53	<0.001
Not booster (N=126)	72 (70–74)	93.46	99.88	<0.001
Not stated (N=3)	62 (42–82)	2.06	-	-
Vaccine rollout				
Before (N=71)	72 (69–75)	52.89	99.90	<0.001
After (N=64)	70 (67–74)	47.11	99.71	<0.001
Sampling methodology				
Non-probability (N=106)	74 (71–76)	78.51	99.83	<0.001
Probability (N=10)	66 (54–77)	7.41	99.48	<0.001
Mixed methods (N=2)	77 (77–77)	1.51	-	-
Not stated (N=17)	59 (50–67)	12.58	99.95	<0.001
Data collection method				
Administered by researchers (N=7)	72 (59–85)	5.05	99.36	<0.001
Not administered by researchers (N=118)	73 (70–75)	87.58	99.85	<0.001
Not stated (N=10)	54 (48–59)	7.37	99.85	<0.001
Questionnaire type				
Likert scale (N=52)	72 (68–76)	38.52	99.81	<0.001
Yes or no (N=64)	72 (68–75)	47.46	99.80	<0.001
Not stated (N=19)	66 (58–73)	14.02	99.95	<0.001
Newcastle Ottawa Scale criteria				
Good (N=67)	72 (68–75)	49.77	99.86	<0.001
Moderate (N=47)	74 (68–79)	34.85	99.78	<0.001

Poor (N=13)	70 (59–80)	9.45	99.78	<0.001
Not available (N=8)	53 (47–60)	5.93	99.94	<0.001
Joanna-Briggs Institute criteria				
Low risk (N=70)	70 (67–74)	51.92	99.84	<0.001
Moderate risk (N=45)	76 (71–80)	33.31	99.78	<0.001
High risk (N=12)	73 (64–83)	8.85	99.76	<0.001
Not available (N=8)	53 (47–60)	5.93	99.94	<0.001

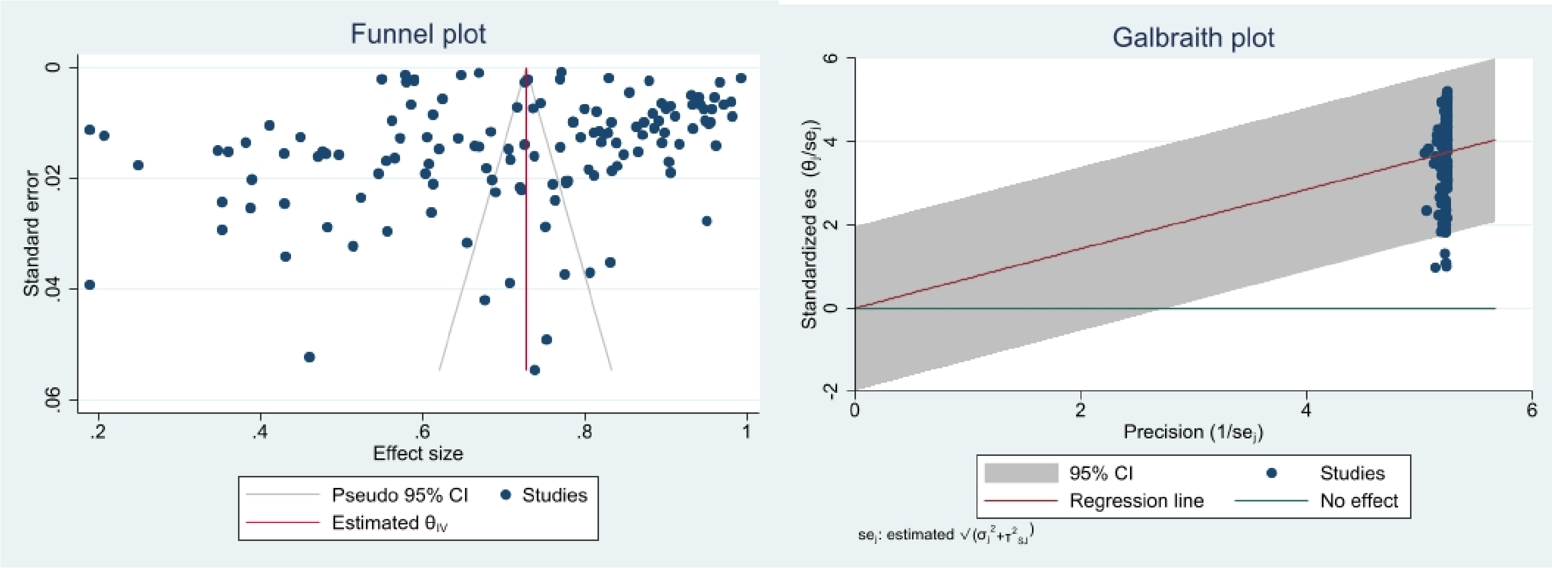

Figure S1. Galbraith plot (left) and funnel plot (right) of COVID-19 vaccine acceptance prevalence.

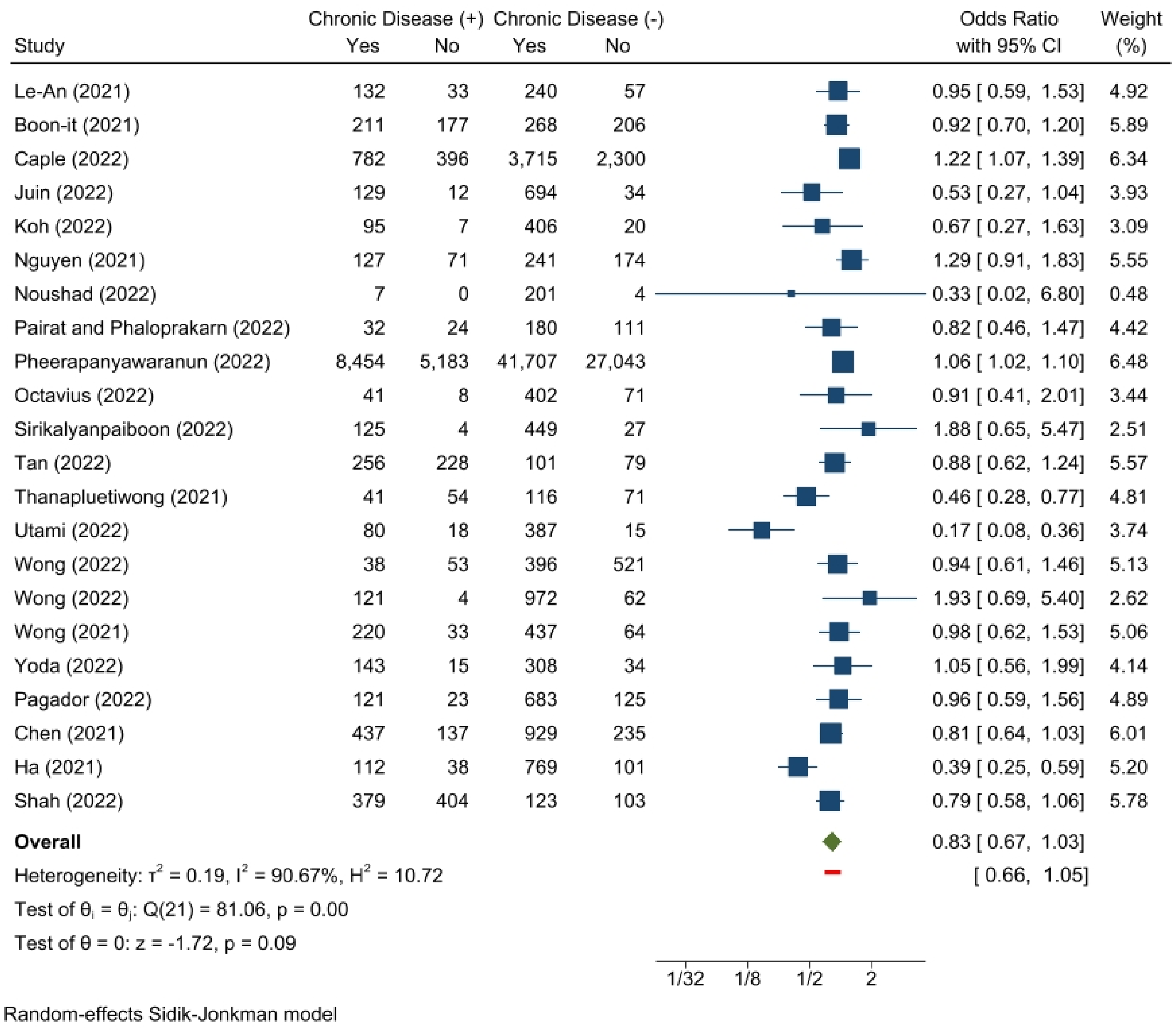

Figure S2. Forest plot of chronic diseases in predicting COVID-19 vaccine acceptance.

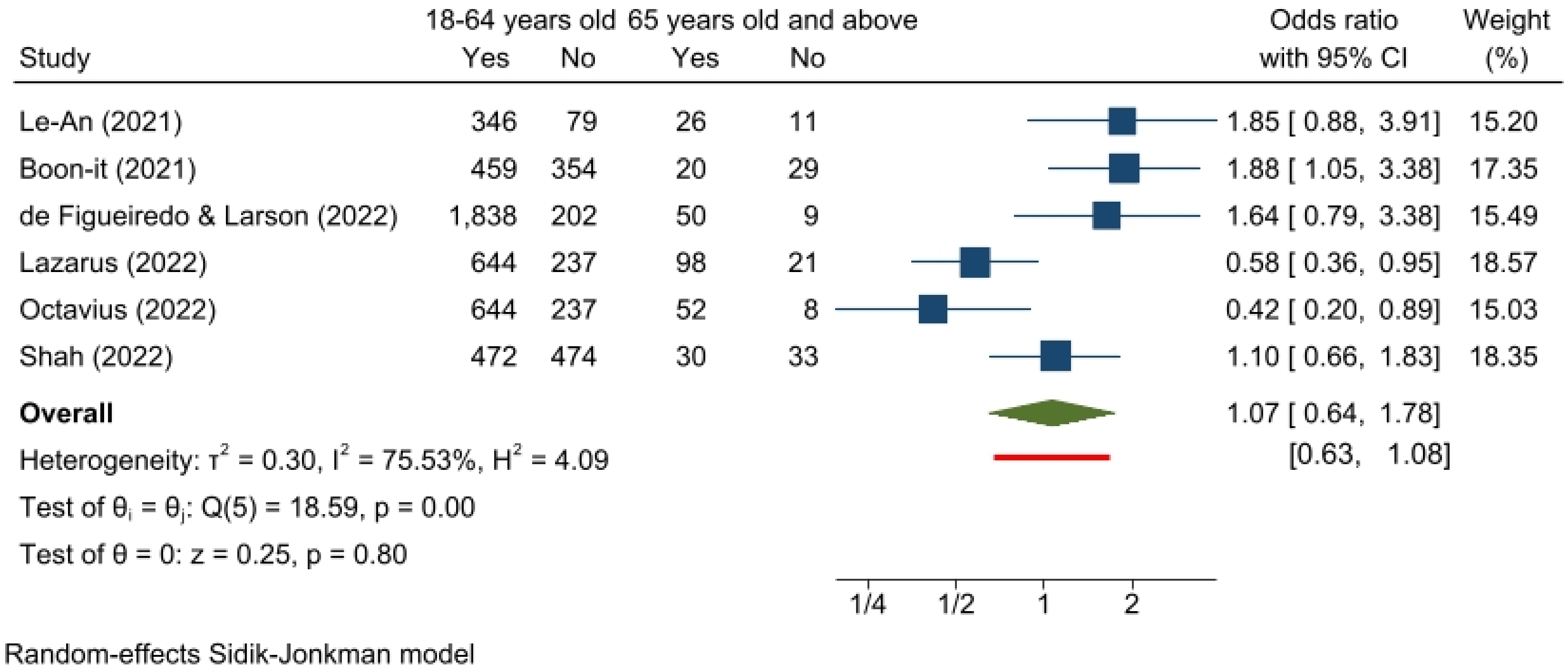

Figure S3. Forest plot of age in predicting COVID-19 vaccine acceptance.

Study	At least completed senior high schools Yes	At least completed senior high schools No	Junior high schools and below Yes	Junior high schools and below No	Odds ratio with 95% CI	Weight (%)
Le-An (2021)	246	57	126	33	1.13 [0.70, 1.83]	7.40
Caple (2022)	4,480	2,666	17	30	2.97 [1.63, 5.39]	7.16
de Figueiredo & Larson (2022)	834	112	1,574	264	1.25 [0.99, 1.58]	7.75
Juin (2022)	784	46	39	0	0.21 [0.01, 3.53]	2.46
Lazarus (2022)	631	228	111	30	0.75 [0.49, 1.15]	7.48
Pairat and Phaloprakarn (2022)	79	39	131	96	1.48 [0.93, 2.36]	7.42
Payaprom (2022)	209	237	696	1,058	1.34 [1.09, 1.65]	7.78
Octavius (2022)	390	65	54	12	1.33 [0.68, 2.63]	6.98
Tan (2022)	404	193	294	114	0.81 [0.62, 1.07]	7.71
Yoda (2022)	432	38	19	11	6.58 [2.92, 14.84]	6.64
Bautista Jr (2021)	93	36	2	4	5.17 [0.91, 29.45]	4.26
Pagador (2022)	802	135	2	13	38.61 [8.62, 173.03]	4.83
Simanjorang (2021)	102	356	20	79	1.13 [0.66, 1.94]	7.28
Ichsan (2021)	42	63	24	77	2.14 [1.17, 3.91]	7.15
Shah (2022)	423	423	107	116	1.08 [0.81, 1.46]	7.69
**Overall**					1.72 [1.01, 2.92]	
					[0.97, 3.04]	

Heterogeneity: $\tau^2 = 0.93$, $I^2 = 95.33\%$, $H^2 = 21.40$

Test of $\theta_i = \theta_j$: $Q(14) = 68.35$, $p = 0.00$

Test of $\theta = 0$: $z = 2.00$, $p = 0.05$

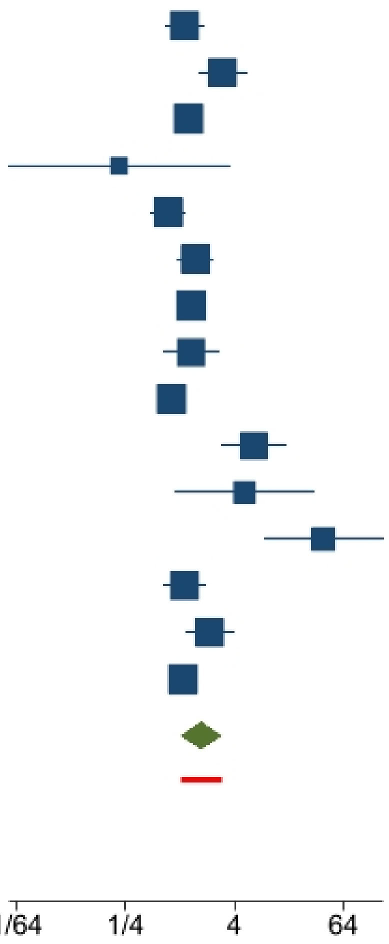

Random-effects Sidik-Jonkman model

Figure S4. Forest plot of education in predicting COVID-19 vaccine acceptance.

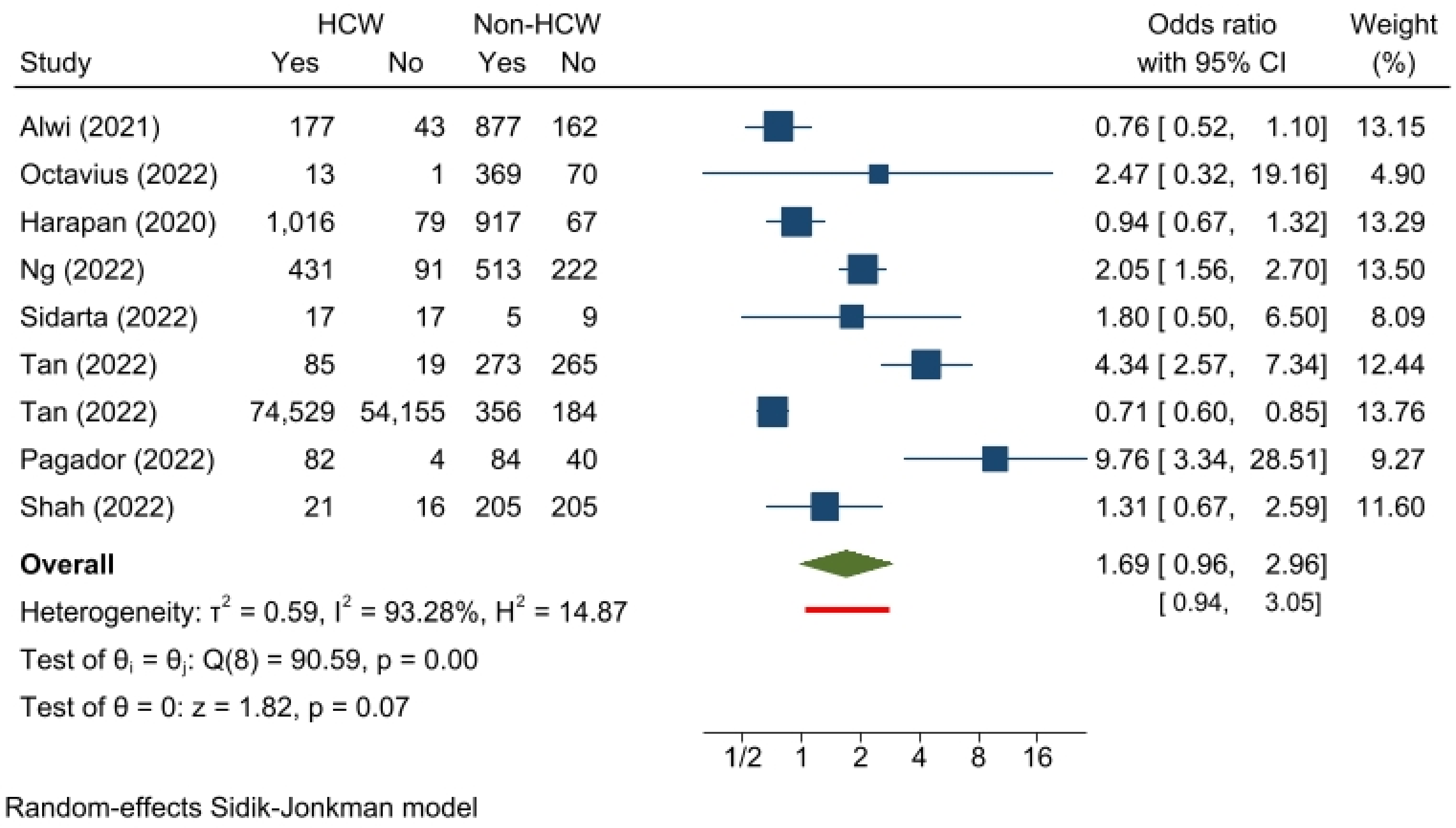

Figure S5. Forest plot of healthcare workers in predicting COVID-19 vaccine acceptance.

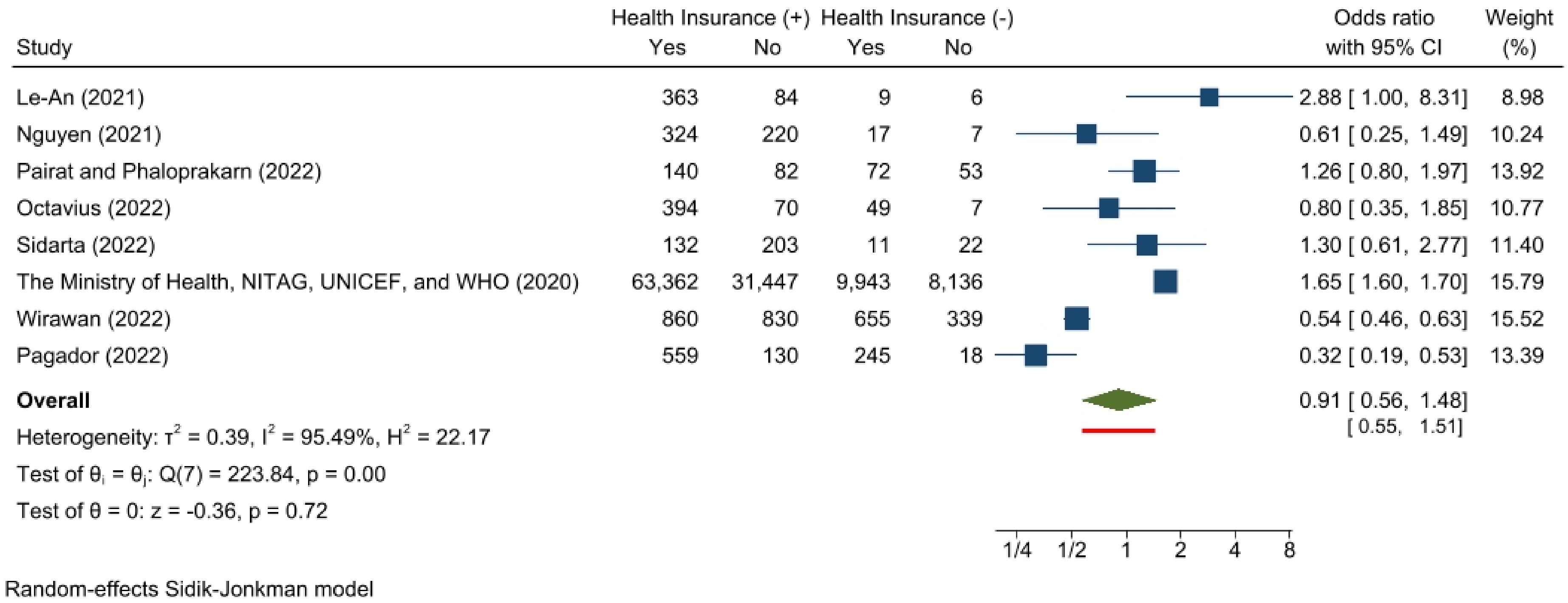

Figure S6. Forest plot of having health insurance in predicting COVID-19 vaccine acceptance.

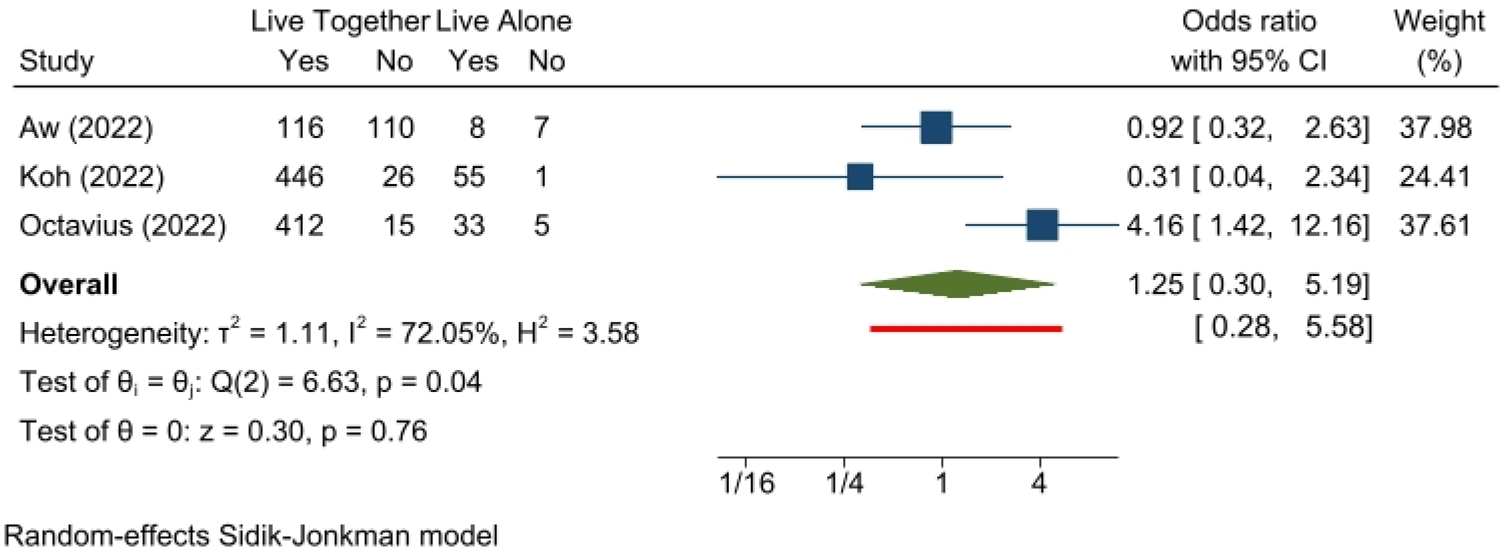

Figure S7. Forest plot of living together in predicting COVID-19 vaccine acceptance.

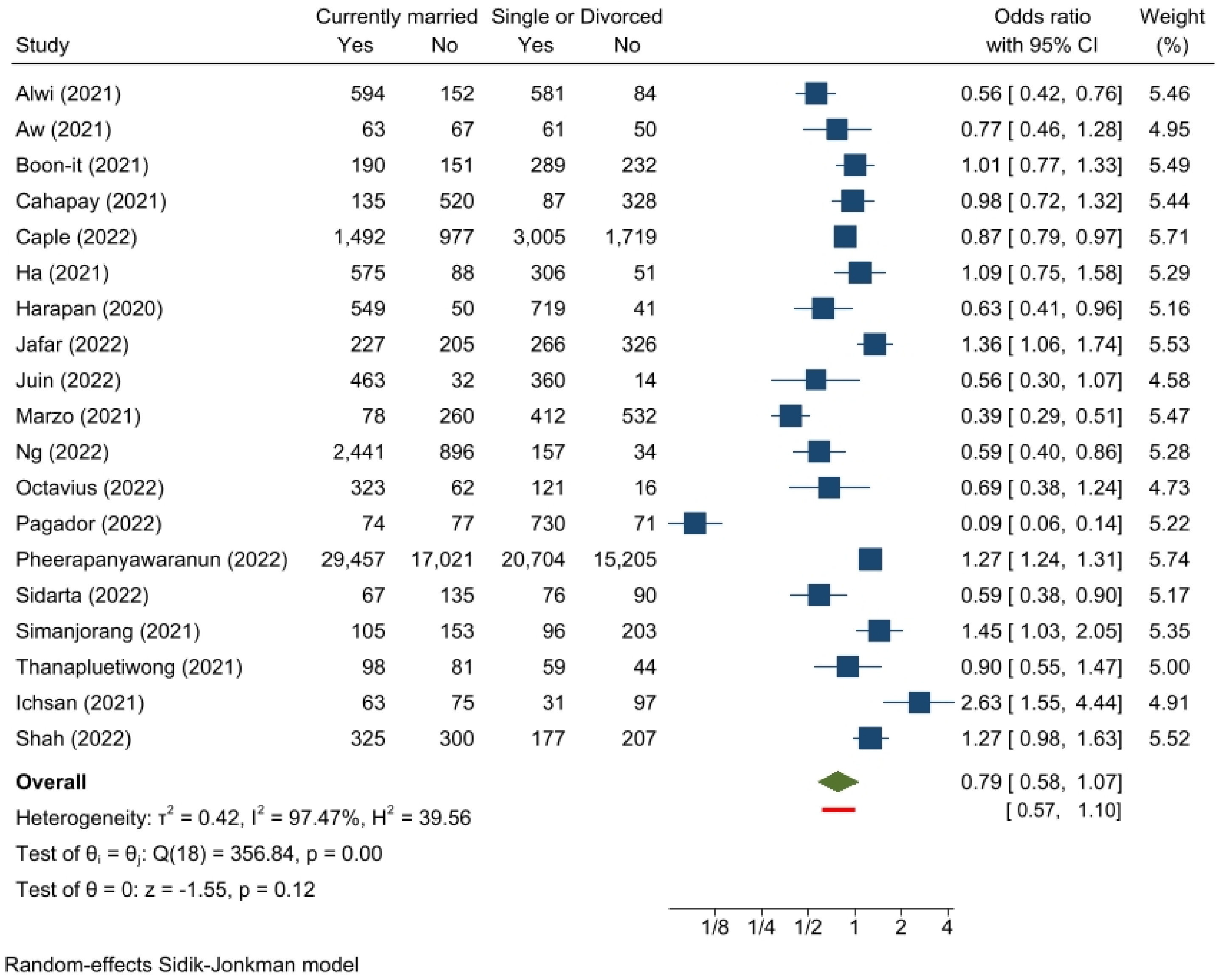

Figure S8. Forest plot of being married in predicting COVID-19 vaccine acceptance.

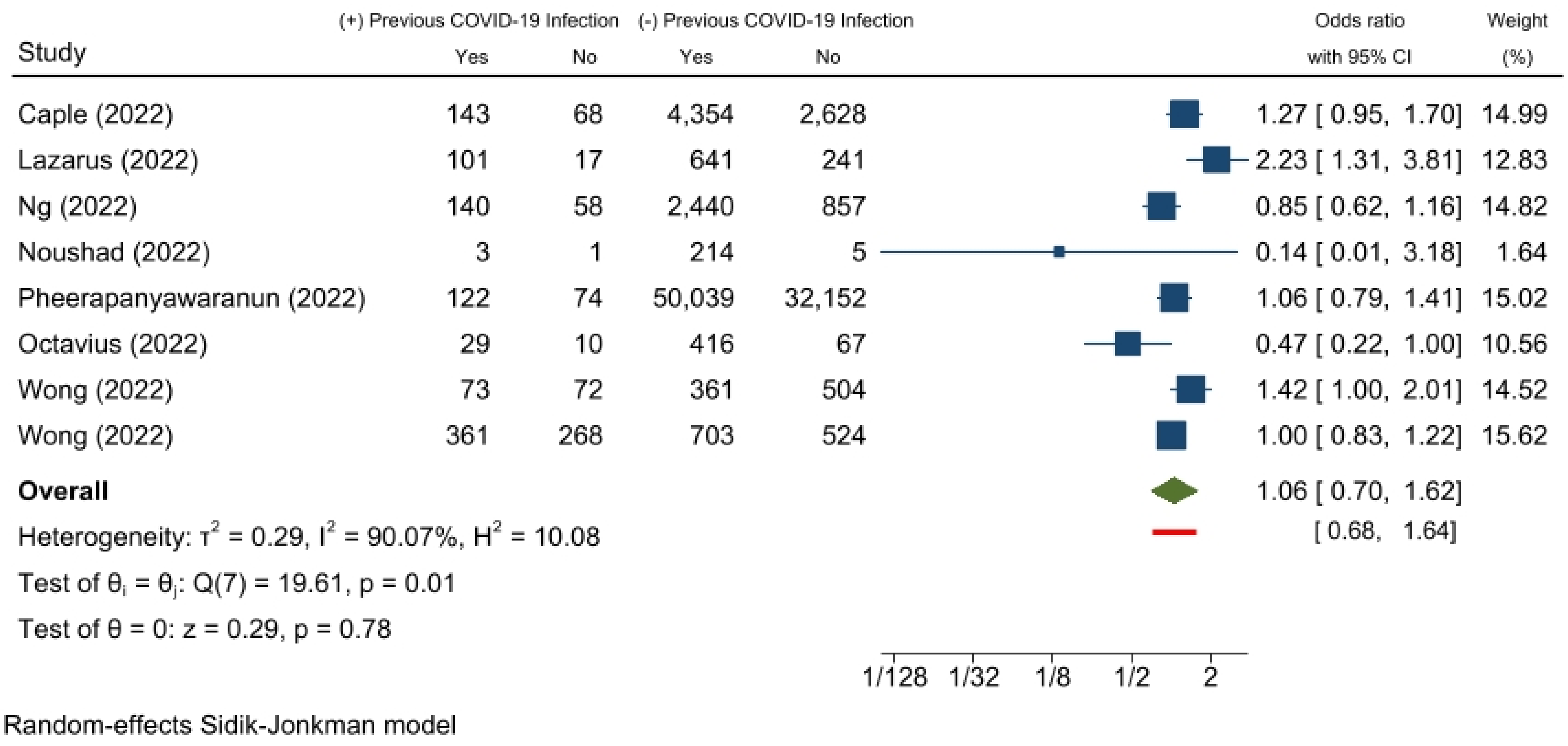

Figure S9. Forest plot of previous COVID-19 infection in predicting COVID-19 vaccine acceptance.

Study	Male Yes	Male No	Female Yes	Female No	Odds ratio with 95% CI	Weight (%)
Alwi (2022)	435	90	740	146	0.95 [0.71, 1.27]	2.62
Le-An (2021)	189	45	183	45	1.03 [0.65, 1.64]	2.24
Le-An (2021)	251	65	407	131	1.24 [0.89, 1.74]	2.52
Ardiningsih & Kardiwinata (2021)	80	4	97	3	0.62 [0.13, 2.85]	0.66
Aw (2022)	35	15	89	102	2.67 [1.37, 5.22]	1.77
Boon-it (2021)	190	121	289	262	1.42 [1.07, 1.89]	2.63
Cahapay (2021)	72	144	150	704	2.35 [1.68, 3.27]	2.53
Caple (2022)	1,663	741	2,834	1,955	1.55 [1.40, 1.72]	2.90
de Figueiredo & Larson (2022)	896	136	946	122	0.85 [0.65, 1.10]	2.67
Harapan (2020)	426	40	842	51	0.65 [0.42, 0.99]	2.31
Huynh (2021)	94	34	218	64	0.81 [0.50, 1.31]	2.19
International Organization for Migration (2022)	1,270	714	1,685	655	0.69 [0.61, 0.79]	2.87
Jafar (2022)	227	246	266	285	0.99 [0.77, 1.26]	2.70
Juin (2022)	333	15	490	31	1.40 [0.75, 2.64]	1.85
Koh (2022)	64	1	437	26	3.81 [0.51, 28.55]	0.42
Lazarus (2022)	384	94	354	163	1.88 [1.40, 2.52]	2.61
Le (2022)	143	80	382	300	1.40 [1.03, 1.92]	2.57
Marzo (2021)	343	568	147	224	0.92 [0.72, 1.18]	2.70
Ng (2022)	767	266	1,831	664	1.05 [0.89, 1.23]	2.83
Nguyen (2021)	146	43	174	49	0.96 [0.60, 1.52]	2.23
Noushad (2022)	68	1	133	20	10.23 [1.34, 77.82]	0.42
Pairat and Phaloprakarn (2022)	108	68	104	67	1.02 [0.66, 1.58]	2.31
Pheerapanyawaranun (2022)	10,614	4,512	39,547	27,714	1.65 [1.59, 1.71]	2.94
Octavius (2022)	227	45	216	34	0.79 [0.49, 1.29]	2.19
Sidarta (2022)	55	80	88	145	1.13 [0.73, 1.75]	2.30
Sirikalyanpaiboon (2022)	333	13	341	18	1.35 [0.65, 2.80]	1.65
Tan (2022)	242	172	105	135	1.81 [1.31, 2.49]	2.55
Tan (2022)	273	108	425	199	1.18 [0.90, 1.56]	2.64
Thanapluetiwong (2021)	50	32	107	93	1.36 [0.80, 2.29]	2.10
The Ministry of Health, NITAG, UNICEF, and WHO (2020)	37,861	20,567	34,547	18,602	0.99 [0.97, 1.02]	2.94
Utami (2022)	97	8	370	25	0.82 [0.36, 1.87]	1.47
Wirawan (2022)	621	502	884	667	0.93 [0.80, 1.09]	2.84
Wong (2022)	152	215	282	361	0.91 [0.70, 1.17]	2.68
Wong (2022)	371	23	722	43	0.96 [0.57, 1.62]	2.10
Wong (2021)	320	34	327	63	1.81 [1.16, 2.83]	2.28
Wong (2022)	401	303	663	489	0.98 [0.81, 1.18]	2.80
Yoda (2022)	234	12	214	37	3.37 [1.71, 6.63]	1.76
Bautista Jr (2021)	33	12	64	26	1.12 [0.50, 2.49]	1.51
Pagador (2022)	287	88	517	60	0.38 [0.26, 0.54]	2.47
Penang Institute (2021)	207	56	102	46	1.67 [1.06, 2.63]	2.25
Chen (2021)	473	127	893	245	1.02 [0.80, 1.30]	2.71
Ha (2021)	266	68	615	71	0.45 [0.31, 0.65]	2.47
Ichsan (2021)	33	52	61	120	1.25 [0.73, 2.13]	2.07
Shah (2022)	254	241	248	266	1.13 [0.88, 1.45]	2.70
**Overall**					1.13 [0.98, 1.31]<br>[0.96, 1.34]	

Heterogeneity: τ^2 = 0.18, I^2 = 96.49%, H^2 = 28.49

Test of $\theta_i = \theta_j$: Q(43) = 746.84, p = 0.00

Test of θ = 0: z = 1.74, p = 0.08

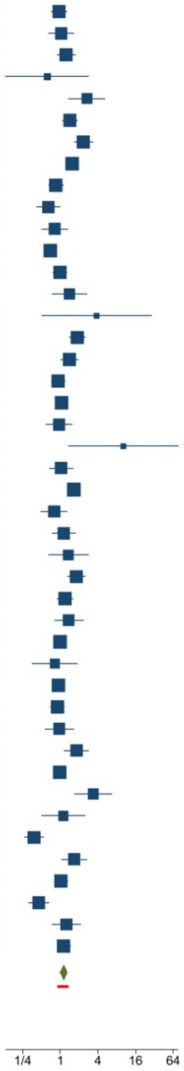

Random-effects Sidik-Jonkman model

Figure S10. Forest plot of being male in predicting COVID-19 vaccine acceptance.

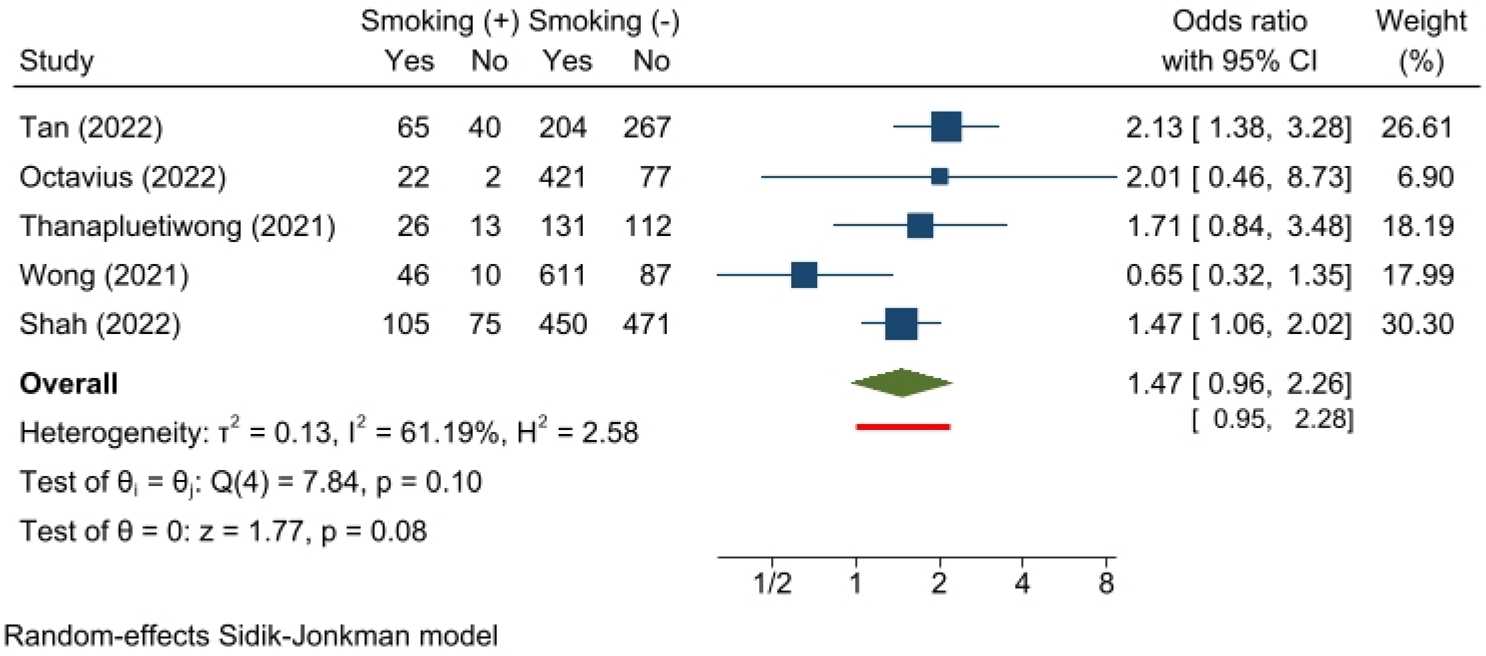

Figure S11. Forest plot of being a smoker in predicting COVID-19 vaccine acceptance.

References

1. Nugraha, R.R.; Miranda, A.V.; Ahmadi, A.; Lucero-Prisno, D.E. Accelerating Indonesian COVID-19 vaccination rollout: a critical task amid the second wave. *Trop. Med. Health* **2021**, *49*, 76, doi:10.1186/s41182-021-00367-3.
2. Suah, J.L.; Tok, P.S.K.; Ong, S.M.; Husin, M.; Tng, B.H.; Sivasampu, S.; Thevananthan, T.; Appannan, M.R.; Muhamad Zin, F.; Mohd Zin, S.; et al. PICK-ing Malaysia's Epidemic Apart: Effectiveness of a Diverse COVID-19 Vaccine Portfolio. *Vaccines (Basel)* **2021**, *9*, doi:10.3390/vaccines9121381.
3. Ng, O.T.; Marimuthu, K.; Lim, N.; Lim, Z.Q.; Thevasagayam, N.M.; Koh, V.; Chiew, C.J.; Ma, S.; Koh, M.; Low, P.Y.; et al. Analysis of COVID-19 Incidence and Severity Among Adults Vaccinated With 2-Dose mRNA COVID-19 or Inactivated SARS-CoV-2 Vaccines With and Without Boosters in Singapore. *JAMA Netw. Open* **2022**, *5*, e2228900-e2228900, doi:10.1001/jamanetworkopen.2022.28900.
4. Pheerapanyawaranun, C.; Wang, Y.; Kittibovorndit, N.; Pimsarn, N.; Sirison, K.; Teerawattananon, Y.; Isaranuwatchai, W. COVID-19 Vaccine Hesitancy Among Health Care Workers in Thailand: The Comparative Results of Two Cross-Sectional Online Surveys Before and After Vaccine Availability. *Front. Public Health* **2022**, *10*, 834545, doi:10.3389/fpubh.2022.834545.

5. Conmigo, V. COVID-19 vaccination roll out among Southern Philippines Medical Center health care workers. *Southern Philippines Med. Cent. J. Health Care Serv.* **2021**, *7*, 4.
6. Mathieu, E.; Ritchie, H.; Ortiz-Ospina, E.; Roser, M.; Hasell, J.; Appel, C.; Giattino, C.; Rodés-Guirao, L. A global database of COVID-19 vaccinations. *Nat. Hum. Behav.* **2021**, *5*, 947-953, doi:10.1038/s41562-021-01122-8.
7. The Asia Foundation. Timor-Leste Covid-19 Survey Round 5 – February 2021. Available online: https://asiafoundation.org/publication/timor-leste-covid-19-survey-round-5-february-2021/ (accessed on 17 September 2022)
8. Nguyen, L.H.; Hoang, M.T.; Nguyen, L.D.; Ninh, L.T.; Nguyen, H.T.T.; Nguyen, A.D.; Vu, L.G.; Vu, G.T.; Doan, L.P.; Latkin, C.A.; et al. Acceptance and willingness to pay for COVID-19 vaccines among pregnant women in Vietnam. *Trop. Med. Int. Health* **2021**, *26*, 1303-1313, doi:https://doi.org/10.1111/tmi.13666.
9. Ardiningsih, N.N.A.; Kardiwinata, M.P. Studi Cross-Sectional: Persepsi Masyarakat Terhadap Penerimaan Vaksinasi COVID-19 di Kabupaten Karangasem. *J. Ris. Kesehat. Nas.* **2021**, *5*, 150-158.
10. Aw, J.; Seah, S.S.Y.; Seng, B.J.J.; Low, L.L. COVID-19-Related Vaccine Hesitancy among Community Hospitals' Healthcare Workers in Singapore. *Vaccines (Basel)* **2022**, *10*, doi:10.3390/vaccines10040537.
11. Bautista Jr, A.P.; Bleza, D.G.; Balibrea, D.M.; Equiza, C. Acceptability of Vaccination Against COVID-19 Among Selected Residents of the Cities of Caloocan, Malabon, and Navotas, Philippines. *Preprints* **2021**, *2021040702* doi:10.20944/preprints202104.0702.v1.
12. Bono, S.A.; Faria de Moura Villela, E.; Siau, C.S.; Chen, W.S.; Pengpid, S.; Hasan, M.T.; Sessou, P.; Ditekemena, J.D.; Amodan, B.O.; Hosseinipour, M.C.; et al. Factors Affecting COVID-19 Vaccine Acceptance: An International Survey among Low- and Middle-Income Countries. *Vaccines (Basel)* **2021**, *9*, doi:10.3390/vaccines9050515.
13. Boon-Itt, S.; Rompho, N.; Jiarnkamolchurn, S.; Skunkan, Y. Interaction between age and health conditions in the intention to be vaccinated against COVID-19 in Thailand. *Hum. Vaccin. Immunother* **2021**, *17*, 4816-4822, doi:10.1080/21645515.2021.1979378.
14. Boontho, J. Factors associated with the decision to receive COVID-19 vaccination among patients with chronic diseases at Somdejprabuddhalertla Hospital, Samutsongkhram province, Thailand. *Dis. Control. J.* **2022**, *48*, 22-32.
15. Cahapay, M.B. To get or not to get: Examining the intentions of Philippine teachers to vaccinate against COVID-19. *J. Hum. Behav. Soc. Environ.* **2022**, *32*, 325-335, doi:10.1080/10911359.2021.1896409.
16. Caple, A.; Dimaano, A.; Sagolili, M.M.; Uy, A.A.; Aguirre, P.M.; Alano, D.L.; Camaya, G.S.; Ciriaco, B.J.; Clavo, P.J.M.; Cuyugan, D.; et al. Interrogating COVID-19 vaccine intent in the Philippines with a nationwide open-access online survey. *Peer.J.* **2022**, *10*, e12887, doi:10.7717/peerj.12887.
17. Chen, W.S.; Siau, C.S.; Bono, S.A.; Low, W.Y. The Influence of Attitudes on the Perceived Effectiveness of the COVID-19 Vaccine in Malaysia. *Asia Pac. J. Public Health* **2022**, *34*, 266-269, doi:10.1177/10105395211065300.
18. Chew, N.W.S.; Cheong, C.; Kong, G.; Phua, K.; Ngiam, J.N.; Tan, B.Y.Q.; Wang, B.; Hao, F.; Tan, W.; Han, X.; et al. An Asia-Pacific study on healthcare workers' perceptions of, and willingness to receive, the COVID-19 vaccination. *Int. J. Infect. Dis.* **2021**, *106*, 52-60, doi:https://doi.org/10.1016/j.ijid.2021.03.069.

19. Crespo, R.F.; Shafat, M.; Melas-Kyriazi, N.; Gould, L.; Jones, S.; Neves, A.L.; Leis, M.S.; Maheswaran, H.; Darzi, A. International attitudes on COVID-19 vaccination: repeat national cross-sectional surveys across 15 countries. *medRxiv* **2021**, 2021.2003.2008.21252449, doi:10.1101/2021.03.08.21252449.
20. Davis, T.P., Jr.; Yimam, A.K.; Kalam, M.A.; Tolossa, A.D.; Kanwagi, R.; Bauler, S.; Kulathungam, L.; Larson, H. Behavioural Determinants of COVID-19-Vaccine Acceptance in Rural Areas of Six Lower- and Middle-Income Countries. *Vaccines (Basel)* **2022**, *10*, doi:10.3390/vaccines10020214.
21. de Figueiredo, A.; Larson, H.J. Exploratory study of the global intent to accept COVID-19 vaccinations. *Commun. Med.* **2021**, *1*, 30, doi:10.1038/s43856-021-00027-x.
22. Duong, M.C.; Nguyen, H.T.; Duong, B.T. Who Influences the Public Intention to Get a COVID-19 Vaccine and What are the Public References and Concerns? A Population Survey in Vietnam. *Infect. Chemother.* **2021**, *53*, 753-766, doi:10.3947/ic.2021.0122.
23. Elnaem, M.H.; Mohd Taufek, N.H.; Ab Rahman, N.S.; Mohd Nazar, N.I.; Zin, C.S.; Nuffer, W.; Turner, C.J. COVID-19 Vaccination Attitudes, Perceptions, and Side Effect Experiences in Malaysia: Do Age, Gender, and Vaccine Type Matter? *Vaccines (Basel)* **2021**, *9*, doi:10.3390/vaccines9101156.
24. Enea, V.; Eisenbeck, N.; Carreno, D.F.; Douglas, K.M.; Sutton, R.M.; Agostini, M.; Bélanger, J.J.; Gützkow, B.; Kreienkamp, J.; Abakoumkin, G.; et al. Intentions to be Vaccinated Against COVID-19: The Role of Prosociality and Conspiracy Beliefs across 20 Countries. *Health Commun.* **2022**, 1-10, doi:10.1080/10410236.2021.2018179.
25. Ginting, D.; Fentiana, N.; Dachi, R.A. Survei Cross-Sectional Online Untuk Menilai Vaksin Covid-19 Terkait Akseptabilitas, Pengetahuan dan Kesediaan Membayar di Kalangan Mahasiswa Kesehatan Masyarakat di Provinsi Sumatera Utara. *J. Ilm. Univ. Batanghari Jambi* **2021**, *21*, 1168-1172.
26. Griva, K.; Tan, K.Y.K.; Chan, F.H.F.; Periakaruppan, R.; Ong, B.W.L.; Soh, A.S.E.; Chen, M.I. Evaluating Rates and Determinants of COVID-19 Vaccine Hesitancy for Adults and Children in the Singapore Population: Strengthening Our Community's Resilience against Threats from Emerging Infections (SOCRATEs) Cohort. *Vaccines (Basel)* **2021**, *9*, doi:10.3390/vaccines9121415.
27. Hà, N.T.; Lăng, N.V.; Dũng, Đ.H.; Thư, P.T.Y. MỘT SỐ YẾU TỐ LIÊN QUAN ĐẾN CHẤP NHẬN TIÊM VẮC XIN PHÒNG COVID-19 DO VIỆT NAM SẢN XUẤT. *Tạp Chí Y học Việt Nam* **2021**, *510*, 249-254.
28. Hadiwijaya, C.R.; Analuddin, M.I.Z.Z.; Sudayasa, A.; Akbar, M.H.; Ahmad, L.A.; Saimin, J. Acceptance of the COVID-19 vaccine in Generation Z: A cross-sectional study in Southeast Sulawesi, Indonesia. *Public Health Indones. 2021 7*, 139-144.
29. Halu, S.A.N.; Dafiq, N.; Banul, M.S.; Lapput, D.O.; Trisnawati, R.E. Factors Affecting Willingness to Vaccinate COVID-19 in Pregnant Women in Manggarai Regency, East Nusa Tenggara, Indonesia. *J. Matern. Child Health* **2022**, *7*, 446-453.
30. Hanvivattanakul, S.; Jongmekwamsuk, K.; Vanichanan, J.; Khawcharoenporn, T. Knowledge and Attitude Toward COVID-19 and Vaccine Acceptance among Health Sciences and Non-Health Sciences Students from Two Large Public Universities in Thailand. *Asian Med. J. Altern. Med.* **2022**, doi:https://doi.org/10.14456/2022s10721.
31. Harapan, H.; Wagner, A.L.; Yufika, A.; Winardi, W.; Anwar, S.; Gan, A.K.; Setiawan, A.M.; Rajamoorthy, Y.; Sofyan, H.; Mudatsir, M. Acceptance of a COVID-19 Vaccine in Southeast Asia: A Cross-Sectional Study in Indonesia. *Front. Public Health* **2020**, *8*, 381, doi:10.3389/fpubh.2020.00381.
32. Hartigan-Go, K.Y.; Mendoza, R.U.; Ong, M.M.A.; Yap, J.K. COVID-19 Vaccine Hesitancy in ASEAN: Insights from a Multi-wave Survey Database from July 2020 to March 2021. *Acta. Medica. Philipp.* **2021**, 1-7.

33. Humanity & Inclusion. Unequal access to Covid-19 vaccines. Available online: https://www.hi-us.org/news-hi-laos-unequal-access-covid19-vaccines (accessed on17 September 2022)
34. Huynh, G.; Tran, T.T.; Nguyen, H.T.N.; Pham, L.A. COVID-19 vaccination intention among healthcare workers in Vietnam. *Asian Pac. J. Trop. Med.* **2021**, *14*, 159-164.
35. Huynh, G.; Nguyen, T.V.; Nguyen, D.D.; Lam, Q.M.; Pham, T.N.; Nguyen, H.T.N. Knowledge About COVID-19, Beliefs and Vaccination Acceptance Against COVID-19 Among High-Risk People in Ho Chi Minh City, Vietnam. *Infect. Drug Resist.* **2021**, *14*, 1773-1780, doi:10.2147/idr.S308446.
36. Ichsan, D.S.; Hafid, F.; Ramadhan, K.; Taqwin. Determinan Kesediaan Masyarakat Menerima Vaksinasi Covid-19 di Sulawesi Tengah. *Poltekita: J. Ilmu Kesehat.* **2021**, *15*, 1-11.
37. International Organization for Migration. Thailand — COVID-19 Vaccine Perceptions of Cambodian and Myanmar Migrants in Thailand (April 2022). Available online: https://dtm.iom.int/reports/thailand-%E2%80%94-covid-19-vaccine-perceptions-cambodian-and-myanmar-migrants-thailand-april-2022 (accessed on17 September 2022)
38. Jafar, A.; Dambul, R.; Dollah, R.; Sakke, N.; Mapa, M.T.; Joko, E.P. COVID-19 vaccine hesitancy in Malaysia: Exploring factors and identifying highly vulnerable groups. *PLOS ONE* **2022**, *17*, e0270868, doi:10.1371/journal.pone.0270868.
39. Juin, J.C.Y.; Ern, S.L.S.; Min, C.; Jing, N.K.; Qi, M.N.M.; Hoe, R.C.C.; Ling, T.C.X.; Yi, F.F.J.; Germain, G.S.L.; Venkatesh, K.N.S.; et al. Knowledge, Attitudes, and Practices of COVID-19 Vaccination among Adults in Singapore: A Cross-Sectional Study. *Am. J. Trop. Med. Hyg.* **2022**, doi:10.4269/ajtmh.21-1259.
40. Jukkrit W.; Nattapong A.; Nuttida K. Knowledge, Attitude, Practice and Acceptance of COVID-19 Vaccine among Elderly in Chiang Mai, Thailand. *J. Educ. Community Health* **2021**, *8*, 245-251.
41. Kementerian Kesihatan Malaysia. Hasil tinjauan vaksin #COVID19: Terima atau tolak? Available online: https://twitter.com/KKMPutrajaya/status/1344580831206023169?s=20 (accessed on 17 September 2022)
42. Kerekes, S.; Ji, M.; Shih, S.-F.; Chang, H.-Y.; Harapan, H.; Rajamoorthy, Y.; Singh, A.; Kanwar, S.; Wagner, A.L. Differential Effect of Vaccine Effectiveness and Safety on COVID-19 Vaccine Acceptance across Socioeconomic Groups in an International Sample. *Vaccines (Basel)* **2021**, *9*, doi:10.3390/vaccines9091010.
43. Khuc, Q.V.; Nguyen, T.; Nguyen, T.; Pham, L.; Le, D.-T.; Ho, H.-H.; Truong, T.-B.; Tran, Q.-K. Young Adults' Intentions and Rationales for COVID-19 Vaccination Participation: Evidence from a Student Survey in Ho Chi Minh City, Vietnam. *Vaccines (Basel)* **2021**, *9*, doi:10.3390/vaccines9070794.
44. Khuc, Q.V. Determinants of COVID-19 vaccine hesitancy and implications for improving vaccination strategy and public health policy. **2021**.
45. Kitro, A.; Sirikul, W.; Piankusol, C.; Rirermsoonthorn, P.; Seesen, M.; Wangsan, K.; Assavanopakun, P.; Surawattanasakul, V.; Kosai, A.; Sapbamrer, R. Acceptance, attitude, and factors affecting the intention to accept COVID-19 vaccine among Thai people and expatriates living in Thailand. *Vaccine* **2021**, *39*, 7554-7561, doi:10.1016/j.vaccine.2021.11.031.

46. Kitro, A.; Sirikul, W.; Dilokkhamaruk, E.; Sumitmoh, G.; Pasirayut, S.; Wongcharoen, A.; Panumasvivat, J.; Ongprasert, K.; Sapbamrer, R. COVID-19 vaccine hesitancy and influential factors among Thai parents and guardians to vaccinate their children. *Vaccine X* **2022**, *11*, 100182, doi:10.1016/j.jvacx.2022.100182.
47. Koesnoe, S.; Siddiq, T.H.; Pelupessy, D.C.; Yunihastuti, E.; Awanis, G.S.; Widhani, A.; Karjadi, T.H.; Maria, S.; Hasibuan, A.S.; Rengganis, I.; et al. Using Integrative Behavior Model to Predict COVID-19 Vaccination Intention among Health Care Workers in Indonesia: A Nationwide Survey. *Vaccines (Basel)* **2022**, *10*, doi:10.3390/vaccines10050719.
48. Koh, S.W.C.; Liow, Y.; Loh, V.W.K.; Liew, S.J.; Chan, Y.H.; Young, D. COVID-19 vaccine acceptance and hesitancy among primary healthcare workers in Singapore. *BMC Prim. Care* **2022**, *23*, 81, doi:10.1186/s12875-022-01693-z.
49. Koh, S.W.C.; Tan, H.M.; Lee, W.H.; Mathews, J.; Young, D. COVID-19 Vaccine Booster Hesitancy among Healthcare Workers: A Retrospective Observational Study in Singapore. *Vaccines (Basel)* **2022**, *10*, doi:10.3390/vaccines10030464.
50. Lansford, J.E.; Rothenberg, W.A.; Yotanyamaneewong, S.; Alampay, L.P.; Al-Hassan, S.M.; Bacchini, D.; Bornstein, M.H.; Chang, L.; Deater-Deckard, K.; Di Giunta, L.; et al. Compliance with Health Recommendations and Vaccine Hesitancy During the COVID Pandemic in Nine Countries. *Prev. Sci.* **2022**, doi:10.1007/s11121-022-01399-9.
51. Lasmita, Y.; Misnaniarti; Idris, H. Analisis Penerimaan Vaksinasi COVID-19 Di Kalangan Masyarakat. *Jurnal Kesehatan Masyarakat Khatulistiwa* **2021**, *9*, 195-204.
52. Lau, J.F.W.; Woon, Y.L.; Leong, C.T.; Teh, H.S. Factors influencing acceptance of the COVID-19 vaccine in Malaysia: a web-based survey. *Osong Public Health Res Perspect* **2021**, *12*, 361-373, doi:10.24171/j.phrp.2021.0085.
53. Lazarus, J.V.; Ratzan, S.C.; Palayew, A.; Gostin, L.O.; Larson, H.J.; Rabin, K.; Kimball, S.; El-Mohandes, A. A global survey of potential acceptance of a COVID-19 vaccine. *Nature Medicine* **2021**, *27*, 225-228, doi:10.1038/s41591-020-1124-9.
54. Lazarus, J.V.; Wyka, K.; White, T.M.; Picchio, C.A.; Rabin, K.; Ratzan, S.C.; Parsons Leigh, J.; Hu, J.; El-Mohandes, A. Revisiting COVID-19 vaccine hesitancy around the world using data from 23 countries in 2021. *Nat Commun* **2022**, *13*, 3801, doi:10.1038/s41467-022-31441-x.
55. Le, A.T.-K.; Pham, T.Q.; Nguyen, L.T.; Pham, T.D.; Ha, N.V. COVID-19 vaccine acceptance and its determinants among Vietnamese teachers: a web-based cross-sectional survey. *AIMS Public Health* **2022**, *9*, 606-617.
56. Le, C.N.; Nguyen, U.T.T.; Do, D.T.H. Predictors of COVID-19 vaccine acceptability among health professions students in Vietnam. *BMC Public Health* **2022**, *22*, 854, doi:10.1186/s12889-022-13236-3.
57. An, P.L.; Nguyen, H.T.N.; Dang, H.T.B.; Huynh, Q.N.H.; Pham, B.D.U.; Huynh, G. Integrating Health Behavior Theories to Predict Intention to Get a COVID-19 Vaccine. *Health Services Insights* **2021**, *14*, 11786329211060130, doi:10.1177/11786329211060130.
58. Le An, P.; Nguyen, H.T.N.; Nguyen, D.D.; Vo, L.Y.; Huynh, G. The intention to get a COVID-19 vaccine among the students of health science in Vietnam. *Human Vaccines & Immunotherapeutics* **2021**, *17*, 4823-4828, doi:10.1080/21645515.2021.1981726.

59. Li, G.; Zhong, Y.; Htet, H.; Luo, Y.; Xie, X.; Wichaidit, W. COVID-19 Vaccine Acceptance and Associated Factors among Unvaccinated Workers at a Tertiary Hospital in Southern Thailand. *Health Services Research and Managerial Epidemiology* **2022**, *9*, 23333928221083057, doi:10.1177/23333928221083057.
60. Lim, L.J.; Lim, A.J.W.; Fong, K.K.; Lee, C.G. Sentiments Regarding COVID-19 Vaccination among Graduate Students in Singapore. *Vaccines (Basel)* **2021**, *9*, doi:10.3390/vaccines9101141.
61. Lin, F.; Chen, X.; Cheng, E.W. Contextualized impacts of an infodemic on vaccine hesitancy: The moderating role of socioeconomic and cultural factors. *Inf Process Manag* **2022**, *59*, 103013, doi:10.1016/j.ipm.2022.103013.
62. Marzo, R.R.; Ahmad, A.; Abid, K.; Khatiwada, A.P.; Ahmed, A.; Kyaw, T.M.; Abidin, I.B.Z.; Srithar, M.; Sinnathamby, S.; Sarvasundram, A.P.; et al. Factors influencing the acceptability of COVID-19 vaccination: A cross-sectional study from Malaysia. *Vacunas* **2022**, *23*, S33-s40, doi:10.1016/j.vacun.2021.07.007.
63. Marzo, R.R.; Ahmad, A.; Islam, M.S.; Essar, M.Y.; Heidler, P.; King, I.; Thiyagarajan, A.; Jermsittiparsert, K.; Songwathana, K.; Younus, D.A.; et al. Perceived COVID-19 vaccine effectiveness, acceptance, and drivers of vaccination decision-making among the general adult population: A global survey of 20 countries. *PLOS Neglected Tropical Diseases* **2022**, *16*, e0010103, doi:10.1371/journal.pntd.0010103.
64. Ministry of Health of Brunei Darussalam. 59% of residents say they are prepared to take COVID-19 vaccine. Available online: https://thescoop.co/2021/02/06/59-of-residents-say-they-are-prepared-to-take-covid-19-vaccine/ (accessed on 17 September 2022)
65. Mohamed, N.A.; Solehan, H.M.; Mohd Rani, M.D.; Ithnin, M.; Che Isahak, C.I. Knowledge, acceptance and perception on COVID-19 vaccine among Malaysians: A web-based survey. *PLOS ONE* **2021**, *16*, e0256110, doi:10.1371/journal.pone.0256110.
66. Ng, J.W.J.; Vaithilingam, S.; Nair, M.; Hwang, L.A.; Musa, K.I. Key predictors of COVID-19 vaccine hesitancy in Malaysia: An integrated framework. *PLoS One* **2022**, *17*, e0268926, doi:10.1371/journal.pone.0268926.
67. Ng, D.-L.-C.; Gan, G.-G.; Chai, C.-S.; Anuar, N.A.B.; Sindeh, W.; Chua, W.-J.; Said, A.B.; Tan, S.-B. The willingness of parents to vaccinate their children younger than 12 years against COVID-19: a cross-sectional study in Malaysia. *BMC Public Health* **2022**, *22*, 1265, doi:10.1186/s12889-022-13682-z.
68. Nguyen, V.T.; Nguyen, M.Q.; Le, N.T.; Nguyen, T.N.H.; Huynh, G. Predictors of Intention to Get a COVID-19 Vaccine of Health Science Students: A Cross-Sectional Study. *Risk Manag Healthc Policy* **2021**, *14*, 4023-4030, doi:10.2147/rmhp.S328665.
69. Nhu, H.V.; Tuyet-Hanh, T.T.; Quang, N.; Linh, T.N.Q.; Tien, T.Q. COVID-19 Vaccine Acceptance in Vietnam: An Online Cross-Sectional Study. *Asia Pac J Public Health* **2022**, *34*, 131-133, doi:10.1177/10105395211053732.
70. Noushad, M.; Rastam, S.; Nassani, M.Z.; Al-Saqqaf, I.S.; Hussain, M.; Yaroko, A.A.; Arshad, M.; Kirfi, A.M.; Koppolu, P.; Niazi, F.H.; et al. A Global Survey of COVID-19 Vaccine Acceptance Among Healthcare Workers. *Front Public Health* **2021**, *9*, 794673, doi:10.3389/fpubh.2021.794673.
71. Tomacruz, S. 46% of adult Filipinos still unwilling to get vaccinated vs COVID-19. Available online: https://www.rappler.com/nation/octa-research-filipinos-covid-19-vaccine-willingness-february-2021/ (accessed on 17 September 2022)
72. CNN Philippines Staff. OCTA survey: Only 25% of Metro Manila respondents willing to get COVID-19 vaccine. Available online: https://www.cnnphilippines.com/news/2021/1/5/COVID-19-vaccine-survey-Metro-Manila-.html (accessed on 17 September 2022)

73. Octavius, G.S.; Yanto, T.A.; Heriyanto, R.S.; Nisa, H.; Ienawi, C.; Pasai, H.E. COVID-19 vaccination acceptance in Jambi City, Indonesia: A single vaccination Center study. *Vacunas* **2022**, doi:10.1016/j.vacun.2022.06.004.
74. Pagador, P.; Pacleb, A.; Ormita, M.J.; Valencia, F.E.; Velasco, D.H.; Josue-Dominguez, R. Acceptance of COVID-19 Vaccine among Unvaccinated Filipinos. *International Journal of Medical Students* **2022**, doi:https://doi.org/10.5195/ijms.2022.1192.
75. Pairat, K.; Phaloprakarn, C. Acceptance of COVID-19 vaccination during pregnancy among Thai pregnant women and their spouses: a prospective survey. *Reproductive Health* **2022**, *19*, 74, doi:10.1186/s12978-022-01383-0.
76. Payaprom, Y.; Tantipong, H.; Manasatchakun, P.; Chandeekeawchakool, S.; Khamchai, S. COVID-19 Vaccine Acceptance from the Perspective of People Living in Northern Thailand: A Mixed Methods Research. *Nursing Journal CMU* **2022**, *49*, 41-54.
77. Prasert, V.; Thavorncharoensap, M.; Vatcharavongvan, P. Acceptance and willingness to pay under the different COVID-19 vaccines: A contingent valuation method. *Research in Social and Administrative Pharmacy* **2022**, doi:https://doi.org/10.1016/j.sapharm.2022.06.001.
78. Puspitasari, D.; Hilmi, S.N.; Novrinda, H. Hubungan Sosiodemografi, Status Kesehatan, dan Theory of Planned Behavior Dengan Pengambilan Keputusan Vaksinasi COVID-19 Pada Ibu Menyusui. *Jurnal Keperawatan Muhammadiyah Bengkulu* **2022**, *10*, 61-72.
79. Putri, K.E.; Wiranti, K.; Ziliwu, Y.S.; Elvita, M.; Frare, D.Y.; Purdani, R.S.; Niman, S. Kecemasan Masyarakat Akan Vaksinasi COVID-19. *Jurnal Keperawatan Jiwa (JKJ): Persatuan Perawat Nasional Indonesia* **2021**, *9*, 539-548.
80. Rani, M.D.M.; Mohamed, N.A.; Solehan, H.M.; Ithnin, M.; Ariffien, A.R.; Isahak, I. Assessment of acceptability of the COVID-19 vaccine based on the health belief model among Malaysians-A qualitative approach. *PLOS ONE* **2022**, *17*, e0269059, doi:10.1371/journal.pone.0269059.
81. Rizki, S.A.; Kurniawan, J.; Budimulia, P.; Sylvanus, P.; Alexandra, A.; Sinaga, T.D.; Kurniawan, A.; Lugito, N.P.H. Knowledge, Attitude, and Practice in Indonesian Health Care Workers Regarding COVID-19. *Asia Pac J Public Health* **2021**, *33*, 662-664, doi:10.1177/10105395211011017.
82. Rozek, L.S.; Jones, P.; Menon, A.; Hicken, A.; Apsley, S.; King, E.J. Understanding Vaccine Hesitancy in the Context of COVID-19: The Role of Trust and Confidence in a Seventeen-Country Survey. *Int J Public Health* **2021**, *66*, 636255, doi:10.3389/ijph.2021.636255.
83. Saida, S.; Zulfadhli, M.; Jurais, M. Analisis Faktor-Faktor Yang Mempengaruhi Vaccine hesitancy (Keragu-Raguan Vaksin) Pada Mahasiswa Di Era Pandemi Covid-19. *Preventif: Jurnal Kesehatan Masyarakat* **2022**, *13*, 144-154.
84. Shah, S.; Gui, H.; Chua, P.E.Y.; Tan, J.B.; Suen, L.K.; Chan, S.W.; Pang, J. Factors associated with COVID-19 vaccination intent in Singapore, Australia and Hong Kong. *Vaccine* **2022**, *40*, 2949-2959, doi:10.1016/j.vaccine.2022.03.062.
85. Sidarta, C.; Kurniawan, A.; Lugito, N.P.H.; Siregar, J.I.; Sungono, V.; Heriyanto, R.S.; Sieto, N.L.; Halim, D.A.; Jodhinata, C.; Rizki, S.A.; et al. The Determinants of COVID-19 Vaccine Acceptance inSumatra. *Jurnal Kesehatan Masyarakat Nasional (National Public Health Journal)* **2022**, *17*, 32-39.
86. Siewchaisakul, P.; Sarakarn, P.; Nanthanangkul, S.; Longkul, J.; Boonchieng, W.; Wungrath, J. Role of literacy, fear and hesitancy on acceptance of COVID-19 vaccine among village health volunteers in Thailand. *PLoS One* **2022**, *17*, e0270023, doi:10.1371/journal.pone.0270023.
87. Simanjorang, C.; Pangandaheng, N.; Tinungki, Y.; Medea, G.P. [The determinants of SARS-CoV-2 vaccine hesitancy in a rural area of an Indonesia-Philippines border island: A mixed-method study]. *Enferm Clin* **2022**, doi:10.1016/j.enfcli.2022.02.001.

88. Sirikalyanpaiboon, M.; Ousirimaneechai, K.; Phannajit, J.; Pitisuttithum, P.; Jantarabenjakul, W.; Chaiteerakij, R.; Paitoonpong, L. COVID-19 vaccine acceptance, hesitancy, and determinants among physicians in a university-based teaching hospital in Thailand. *BMC Infectious Diseases* **2021**, *21*, 1174, doi:10.1186/s12879-021-06863-5.
89. Skjefte, M.; Ngirbabul, M.; Akeju, O.; Escudero, D.; Hernandez-Diaz, S.; Wyszynski, D.F.; Wu, J.W. COVID-19 vaccine acceptance among pregnant women and mothers of young children: results of a survey in 16 countries. *European Journal of Epidemiology* **2021**, *36*, 197-211, doi:10.1007/s10654-021-00728-6.
90. Syed Alwi, S.A.R.; Rafidah, E.; Zurraini, A.; Juslina, O.; Brohi, I.B.; Lukas, S. A survey on COVID-19 vaccine acceptance and concern among Malaysians. *BMC Public Health* **2021**, *21*, 1129, doi:10.1186/s12889-021-11071-6.
91. Tan, L.F.; Huak, C.Y.; Siow, I.; Tan, A.J.; Venugopalan, P.M.; Premkumar, A.; Seetharaman, S.K.; Tan, B.Y.Q. The road to achieving herd immunity: factors associated with Singapore residents' uptake and hesitancy of the COVID-19 vaccination. *Expert Rev Vaccines* **2022**, *21*, 561-567, doi:10.1080/14760584.2022.2021883.
92. Tan, M.; Straughan, P.T.; Cheong, G. Information trust and COVID-19 vaccine hesitancy amongst middle-aged and older adults in Singapore: A latent class analysis Approach. *Soc Sci Med* **2022**, *296*, 114767, doi:10.1016/j.socscimed.2022.114767.
93. Tan, K.Y.K.; Soh, A.S.E.; Ong, B.W.L.; Chen, M.I.; Griva, K. Determining the Prevalence and Correlates of COVID-19 Booster Vaccine Hesitancy in the Singapore Population Following the Completion of the Primary Vaccination Series. *Vaccines (Basel)* **2022**, *10*, doi:10.3390/vaccines10071088.
94. Tan, K.W.A.; Wijaya, L.; Lim, C.T.; Gan, W.H. COVID-19 vaccination acceptance of healthcare workers in Singapore. *Ann Acad Med Singap* **2022**, *51*, 304-308, doi:10.47102/annals-acadmedsg.2021504.
95. Thanapluetiwong, S.; Chansirikarnjana, S.; Sriwannopas, O.; Assavapokee, T.; Ittasakul, P. Factors associated with COVID-19 Vaccine Hesitancy in Thai Seniors. *Patient Prefer Adherence* **2021**, *15*, 2389-2403, doi:10.2147/ppa.S334757.
96. The Asia Foundation. Timor-Leste Covid-19 Survey Round 6 – May 2021. Available online: https://asiafoundation.org/publication/timor-leste-covid-19-survey-round-6-may-2021/ (accessed on 17 September 2022)
97. The Asia Foundation. Timor-Leste Covid-19 Survey Round 7 – June 2021. Available online: https://asiafoundation.org/publication/timor-leste-covid-19-survey-round-7-june-2021/ (accessed on 17 September 2022)
98. The Asia Foundation. Timor-Leste Covid-19 Survey Round 8 – September 2021. Available online: https://asiafoundation.org/publication/timor-leste-covid-19-survey-round-8-september-2021/ (accessed on 17 September 2022)
99. Thi Xuan Hoang, H.; Abu-Odah, H.; Hoang Vu, A.; Van Nguyen, L. Readiness and determinants of Vietnam's general public to receive the COVID-19 Vaccine: a national online cross-sectional study [version 1; peer review: awaiting peer review]. *F1000Research* **2022**, *11*, doi:10.12688/f1000research.122069.1.
100. UNDP Philippines. Trends in COVID-19 Vaccine Acceptance in the Philippines and their Implications on Health Communication. Available online: https://www.undp.org/philippines/publications/trends-covid-19-vaccine-acceptance-philippines-and-their-implications-health-communication (accessed on 17 September 2022)

101. UNICEF. COVID-19 vaccine acceptance survey in Indonesia. Available online: https://www.unicef.org/indonesia/coronavirus/reports/covid-19-vaccine-acceptance-survey-indonesia (accessed on 17 September 2022)
102. Utami, A.; Margawati, A.; Pramono, D.; Nugraheni, A.; Pramudo, S.G. Determinant Factors of COVID-19 Vaccine Hesitancy Among Adult and Elderly Population in Central Java, Indonesia. *Patient Prefer Adherence* **2022**, *16*, 1559-1570, doi:10.2147/ppa.S365663.
103. Vaghefi, N.; Nie, C.P.; Siang, C.n.K. Survey on Attitudes to Covid-19 Vaccination in Penang. Available online: https://penanginstitute.org/publications/issues/survey-on-attitudes-to-covid-19-vaccination-in-penang/ (accessed on 17 September 2022)
104. Wen, J.L.F.; Tho, L.C.; Shien, T.H.; Liang, W.Y. Factors influencing acceptance of COVID-19 vaccination among Malaysian adults. In Proceedings of the National Conference for Clinical Research, 2020.
105. Wirawan, G.B.S.; Mahardani, P.; Cahyani, M.R.K.; Laksmi, N.; Januraga, P.P. Conspiracy beliefs and trust as determinants of COVID-19 vaccine acceptance in Bali, Indonesia: Cross-sectional study. *Pers Individ Dif* **2021**, *180*, 110995, doi:10.1016/j.paid.2021.110995.
106. Wirawan, G.B.; Harjana, N.P.; Nugrahani, N.W.; Januraga, P.P. Health Beliefs and Socioeconomic Determinants of COVID-19 Booster Vaccine Acceptance: An Indonesian Cross-Sectional Study. *Vaccines (Basel)* **2022**, *10*, doi:10.3390/vaccines10050724.
107. Wong, L.P.; Alias, H.; Siaw, Y.L.; Muslimin, M.; Lai, L.L.; Lin, Y.; Hu, Z. Intention to receive a COVID-19 vaccine booster dose and associated factors in Malaysia. *Hum Vaccin Immunother* **2022**, 2078634, doi:10.1080/21645515.2022.2078634.
108. Wong, L.P.; Alias, H.; Wong, P.F.; Lee, H.Y.; AbuBakar, S. The use of the health belief model to assess predictors of intent to receive the COVID-19 vaccine and willingness to pay. *Hum Vaccin Immunother* **2020**, *16*, 2204-2214, doi:10.1080/21645515.2020.1790279.
109. Wong, L.P.; Alias, H.; Danaee, M.; Ahmed, J.; Lachyan, A.; Cai, C.Z.; Lin, Y.; Hu, Z.; Tan, S.Y.; Lu, Y.; et al. COVID-19 vaccination intention and vaccine characteristics influencing vaccination acceptance: a global survey of 17 countries. *Infect Dis Poverty* **2021**, *10*, 122, doi:10.1186/s40249-021-00900-w.
110. Wong, L.P.; Alias, H.; Tan, Y.R.; Tan, K.M. Older people and responses to COVID-19: A cross-sectional study of prevention practices and vaccination intention. *Int J Older People Nurs* **2022**, *17*, e12436, doi:10.1111/opn.12436.
111. Wong, L.P.; Alias, H.; Megat Hashim, M.; Lee, H.Y.; AbuBakar, S.; Chung, I.; Hu, Z.; Lin, Y. Acceptability for COVID-19 vaccination: perspectives from Muslims. *Hum Vaccin Immunother* **2022**, *18*, 2045855, doi:10.1080/21645515.2022.2045855.
112. Wong, E.L.; Qiu, H.; Chien, W.T.; Wong, J.C.; Chalise, H.N.; Hoang, H.T.; Nguyen, H.T.; Chan, P.K.; Wong, M.C.; Cheung, A.W.; et al. COVID-19 Vaccine Willingness and Related Factors Among Health Care Workers in 3 Southeast Asian Jurisdictions. *JAMA Netw Open* **2022**, *5*, e2228061, doi:10.1001/jamanetworkopen.2022.28061.
113. World Vision International. COVID-19 Vaccine Barrier Analysis Survey. Available online: https://www.wvi.org/sites/default/files/2021-05/WVL%20COVID%20Vaccine%20-%20Barrier%20Analysis%20staff_A4.pdf (accessed on 17 September 2022)
114. Yoda, T.; Suksatit, B.; Tokuda, M.; Katsuyama, H. Analysis of People's Attitude Toward COVID-19 Vaccine and Its Information Sources in Thailand. *Cureus* **2022**, *14*, e22215, doi:10.7759/cureus.22215.

115. Bono, S.A.; Siau, C.S.; Chen, W.S.; Low, W.Y.; Faria de Moura Villela, E.; Pengpid, S.; Hasan, M.T.; Sessou, P.; Ditekemena, J.D.; Amodan, B.O.; et al. Adults' Acceptance of COVID-19 Vaccine for Children in Selected Lower- and Middle-Income Countries. *Vaccines (Basel)* **2021**, *10*, doi:10.3390/vaccines10010011.
116. Chen, W.S.; Siau, C.S.; Bono, S.A.; Low, W.Y. Mediating Effect of Chronic Illnesses in the Relationship Between Psychological Distress and COVID-19 Vaccine Acceptance. *Asia Pac J Public Health* **2022**, *34*, 106-112, doi:10.1177/10105395211047868.
117. Duong, M.C.; Duong, B.T.; Nguyen, H.T.; Nguyen Thi Quynh, T.; Nguyen, D.P. Knowledge about COVID-19 vaccine and vaccination in Vietnam: A population survey. *J Am Pharm Assoc (2003)* **2022**, *62*, 1197-1205.e1194, doi:10.1016/j.japh.2022.01.014.
118. Harapan, H.; Wagner, A.L.; Yufika, A.; Winardi, W.; Anwar, S.; Gan, A.K.; Setiawan, A.M.; Rajamoorthy, Y.; Sofyan, H.; Vo, T.Q.; et al. Willingness-to-pay for a COVID-19 vaccine and its associated determinants in Indonesia. *Hum Vaccin Immunother* **2020**, *16*, 3074-3080, doi:10.1080/21645515.2020.1819741.
119. Jafar, A.; Mapa, M.T.; Sakke, N.; Dollah, R.; Joko, E.P.; Atang, C.; Awang Ahmad, S.; Vun Hung, C.; Geogre, F. Vaccine hesitancy in East Malaysia (Sabah): A survey of the national COVID-19 immunisation programme. *Geospat Health* **2022**, *17*, doi:10.4081/gh.2022.1037.
120. Leigh, J.P.; Moss, S.J.; White, T.M.; Picchio, C.A.; Rabin, K.H.; Ratzan, S.C.; Wyka, K.; El-Mohandes, A.; Lazarus, J.V. Factors affecting COVID-19 vaccine hesitancy among healthcare providers in 23 countries. *Vaccine* **2022**, *40*, 4081-4089, doi:https://doi.org/10.1016/j.vaccine.2022.04.097.
121. Marzo, R.R.; Sami, W.; Alam, M.Z.; Acharya, S.; Jermsittiparsert, K.; Songwathana, K.; Pham, N.T.; Respati, T.; Faller, E.M.; Baldonado, A.M.; et al. Hesitancy in COVID-19 vaccine uptake and its associated factors among the general adult population: a cross-sectional study in six Southeast Asian countries. *Tropical Medicine and Health* **2022**, *50*, 4, doi:10.1186/s41182-021-00393-1.
122. Yanto, T.A.; Octavius, G.S.; Heriyanto, R.S.; Ienawi, C.; Nisa, H.; Pasai, H.E. Psychological factors affecting COVID-19 vaccine acceptance in Indonesia. *The Egyptian Journal of Neurology, Psychiatry and Neurosurgery* **2021**, *57*, 177, doi:10.1186/s41983-021-00436-8.
123. Zhang, F.; Shih, S.-F.; Harapan, H.; Rajamoorthy, Y.; Chang, H.-Y.; Singh, A.; Lu, Y.; Wagner, A.L. Changes in COVID-19 risk perceptions: methods of an internet survey conducted in six countries. *BMC Research Notes* **2021**, *14*, 428, doi:10.1186/s13104-021-05846-8.
124. Liew, Z.H.; Leeu, J.J.; Tan, H.Z.; Mok, I.Y.J.; Choo, J.C.J.; Lim, C.C. COVID-19 vaccine acceptance among patients with glomerulonephritis. *Nephrology (Carlton)* **2022**, *27*, 543-545, doi:10.1111/nep.14026.
125. Lim, C.C.; Mok, I.Y.J.; Leeu, J.J.; Liew, Z.H.; Tan, H.Z.; Chin, Y.M.; Teng, W.L.; Yeo, F.; Tan, C.S.; Choo, J.C.J. A Descriptive Evaluation of Health Literacy and Determinants of COVID-19 Vaccine Acceptance among Patients with IgA Nephropathy with High Vaccine Uptake. *Glomerular Diseases* **2022**, *2*, 132-138, doi:10.1159/000522158.
126. Landicho-Guevarra, J.; Reñosa, M.D.C.; Wachinger, J.; Endoma, V.; Aligato, M.F.; Bravo, T.A.; Landicho, J.; Bärnighausen, K.; McMahon, S.A. Scared, powerless, insulted and embarrassed: hesitancy towards vaccines among caregivers in Cavite Province, the Philippines. *BMJ Global Health* **2021**, *6*, e006529, doi:10.1136/bmjgh-2021-006529.

127. Migriño, J., Jr.; Gayados, B.; Birol, K.R.J.; De Jesus, L.; Lopez, C.W.; Mercado, W.C.; Tolosa, J.C.; Torreda, J.; Tulagan, G. Factors affecting vaccine hesitancy among families with children 2 years old and younger in two urban communities in Manila, Philippines. *Western Pac Surveill Response J* **2020**, *11*, 20-26, doi:10.5365/wpsar.2019.10.2.006.
128. Musa, A.; Soni, T.; Cheong, X.; Nordin, R. Vaccine hesitancy among parents in Kuala Lumpur: a single center study [version 1; peer review: 2 approved with reservations, 1 not approved]. *F1000Research* **2019**, *8*, doi:10.12688/f1000research.20079.1.
129. Shaaban, R.; Ghazy, R.M.; Elsherif, F.; Ali, N.; Yakoub, Y.; Aly, M.O.; ElMakhzangy, R.; Abdou, M.S.; McKinna, B.; Elzorkany, A.M.; et al. COVID-19 Vaccine Acceptance among Social Media Users: A Content Analysis, Multi-Continent Study. *Int J Environ Res Public Health* **2022**, *19*, doi:10.3390/ijerph19095737.
130. Duong, A.H.; Antriyandarti, E. COVID-19 Vaccine Acceptance among ASEAN Countries: Does the Pandemic Severity Really Matter? *Vaccines (Basel)* **2022**, *10*, doi:10.3390/vaccines10020222.
131. Faturohman, T.; Kengsiswoyo, G.A.N.; Harapan, H.; Zailani, S.; Rahadi, R.A.; Arief, N.N. Factors influencing COVID-19 vaccine acceptance in Indonesia: an adoption of Technology Acceptance Model. *F1000Res* **2021**, *10*, 476, doi:10.12688/f1000research.53506.2.
132. Leesawat, C.; Detkong, T.; Thanaphakawat, L. Factors affecting COVID-19 vaccine hesitancy and acceptance. *Journal of Mental Health of Thailand* **2022**, *30*, 161-170.
133. Mangla, S.; Zohra Makkia, F.T.; Pathak, A.K.; Robinson, R.; Sultana, N.; Koonisetty, K.S.; Karamehic-Muratovic, A.; Nguyen, U.D.T.; Rodriguez-Morales, A.J.; Sanchez-Duque, J.A.; et al. COVID-19 Vaccine Hesitancy and Emerging Variants: Evidence from Six Countries. *Behav Sci (Basel)* **2021**, *11*, doi:10.3390/bs11110148.
134. Mueangpoon, K.; Inchan, C.; Kaewmuneechoke, P.; Rattana, P.; Budsratid, S.; Japakiya, S.; Ngamchaliew, P.; Vichitkunakorn, P. Self-Reported COVID-19 Vaccine Hesitancy and Willingness to Pay: A Cross-Sectional Survey in Thailand. *Vaccines (Basel)* **2022**, *10*, doi:10.3390/vaccines10040627.
135. Sirait, H.S.; Saidah, Q.; Hasanah, O.; Hanifah, A.N.; Arifin, H.; Rosyad, Y.S.; Rias, Y.A. Indonesian nursing students' intention to accept COVID-19 vaccines: an online, multicentre survey. *Br J Nurs* **2022**, *31*, 488-494, doi:10.12968/bjon.2022.31.9.488.
136. Susilawaty, A.; Noviyanto, F.; Afrianty, L.; Syahputra, A.; Kurniasari, L.; Handoko, L.; Wulandari, R.; Pramana, C. Attitude, Risk Perception and Public Acceptance against Coronavirus Disease 2019 Vaccination in Indonesia. *Open Access Macedonian Journal of Medical Sciences* **2021**, *9*, 717-721.
137. Teh, H.S.; Woon, Y.L.; Leong, C.T.; Hing, N.Y.L.; Mien, T.Y.S.; Roope, L.S.J.; Clarke, P.M.; Lim, L.L.; Buckell, J. Malaysian public preferences and decision making for COVID-19 vaccination: A discrete choice experiment. *Lancet Reg Health West Pac* **2022**, *27*, 100534, doi:10.1016/j.lanwpc.2022.100534.
138. The World Bank. Reducing Vaccine Hesitancy in the Philippines - Findings from a Survey Experiment. Available online: https://thedocs.worldbank.org/en/doc/9b206c064482a4fbb880ee23d6081d52-0070062021/original/Vaccine-Hesitancy-World-Bank-Policy-Note-September-2021.pdf (accessed on 17 September 2022)
139. Theodorea, C.F.; Widyarman, A.S.; Dewanto, I.; Astoeti, T.E. COVID-19 Vaccines in Indonesia: Knowledge, Attitudes, and Acceptance Among Dental Professionals. *Front Med (Lausanne)* **2021**, *8*, 784002, doi:10.3389/fmed.2021.784002.

140. Wee, M.K.; Cabantog, J.; Magpayo, D.D.; Sabido, N.L.; Samson, E.; David, P. Factors causing vaccine hesitancy among parents in Bulacan. *Studied in Medicine and Public Health (SiMPH)* **2021**, *1*, 15-29.
141. Zagefka, H.; dela Paz, E.; Macapagal, M.E.J.; Ghazal, S. Personal willingness to receive a Covid-19 vaccine and its relationship with intergroup psychology: Evidence from the Philippines and Pakistan. *Applied Psychology: Health and Well-Being* **2022**, *n/a*, doi:https://doi.org/10.1111/aphw.12334.

.
